# A myxozoan genome reveals mosaic evolution in a parasitic cnidarian

**DOI:** 10.1186/s12915-022-01249-8

**Published:** 2022-02-18

**Authors:** Qingxiang Guo, Stephen D. Atkinson, Bin Xiao, Yanhua Zhai, Jerri L. Bartholomew, Zemao Gu

**Affiliations:** 1 Department of Aquatic Animal Medicine, College of Fisheries, Huazhong Agricultural University, Wuhan, 430070 People’s Republic of China; 2 Hubei Engineering Technology Research Center for Aquatic Animal Diseases Control and Prevention, Wuhan, 430070 People’s Republic of China; 3 Department of Microbiology, Oregon State University, Corvallis, OR 97331 USA

**Keywords:** Evolutionary genomics, Parasite evolution, Genome streamlining, Cnidaria, Myxozoa, *Myxobolus honghuensis*

## Abstract

**Background:**

Parasite evolution has been conceptualized as a process of genetic loss and simplification. Contrary to this model, there is evidence of expansion and conservation of gene families related to essential functions of parasitism in some parasite genomes, reminiscent of widespread *mosaic evolution*—where subregions of a genome have different rates of evolutionary change. We found evidence of mosaic genome evolution in the cnidarian *Myxobolus honghuensis*, a myxozoan parasite of fish, with extremely simple morphology.

**Results:**

We compared *M. honghuensis* with other myxozoans and free-living cnidarians, and determined that it has a relatively larger myxozoan genome (206 Mb), which is less reduced and less compact due to gene retention, large introns, transposon insertion, but not polyploidy. Relative to other metazoans, the *M. honghuensis* genome is depleted of neural genes and has only the simplest animal immune components. Conversely, it has relatively more genes involved in stress resistance, tissue invasion, energy metabolism, and cellular processes compared to other myxozoans and free-living cnidarians. We postulate that the expansion of these gene families is the result of evolutionary adaptations to endoparasitism. *M. honghuensis* retains genes found in free-living Cnidaria, including a reduced nervous system, myogenic components, ANTP class Homeobox genes, and components of the Wnt and Hedgehog pathways.

**Conclusions:**

Our analyses suggest that the *M. honghuensis* genome evolved as a mosaic of conservative, divergent, depleted, and enhanced genes and pathways. These findings illustrate that myxozoans are not as genetically simple as previously regarded, and the evolution of some myxozoans is driven by both genomic streamlining and expansion.

**Supplementary Information:**

The online version contains supplementary material available at 10.1186/s12915-022-01249-8.

## Background

Parasite evolution is a focal issue in evolutionary biology and ecology [1, 2]. Understanding the evolutionary processes of the transition of ancestral free-living organisms to parasitism is important for human health and agriculture and offers a new testing ground for evolutionary and ecological theory [3, 4]. Relative to free-living species, parasite genomes are typically smaller and more tightly packed with protein-coding genes [5, 6]. Parasite evolution is usually considered to be accompanied by loss of genetic complexity, as exploitation of host metabolic pathways releases selective constraints on parts of the parasite genome, resulting in loss of now-redundant functions (and associated genes) [7]. Accordingly, an appealing reductive theory of evolution has been developed to describe this loss of function and subsequent streamlining that governs parasite genome evolution [8, 9]. However, there is evidence for conservation and even expansion of gene families associated with infection and survival in some parasite genomes [6, 10]. The opposing characters of genomic streamlining and expansion are components of a pervasive process known as mosaic evolution, which is the tendency for a genome to evolve as a set of discrete units, each with its own evolutionary mode, rather than being dominated by a uniform trend [11, 12]. Mosaic evolution is widely used to frame observed genomic changes in free-living taxa including mice, humans, and birds [12–15]. There is much less evidence of selective genetic expansion in parasites [6, 10, 16–18]; thus, mosaic evolution has rarely been emphasized in describing parasite evolutionary patterns. We propose that a more complete picture of parasite genome evolution should incorporate the opposing features of streamlining and novel complexity.

Cnidarians are early-diverging metazoans and ideally suited for investigating the genomic changes that underlie parasitism, as the group includes both free-living (e.g., Medusozoa, Anthozoa) and parasitic taxa (Myxozoa and Polypodiozoa) [19]. Myxozoa are microscopic, oligocellular endoparasites with simple body organization, but multiple morphologies in their complex life cycles [20, 21]. The few sequenced myxozoan genomes are more compact and smaller than those of free-living cnidarians [22], with some species having reduced metabolic capacity [23], or having lost core animal features such as cytosine methylation [24] or their mitochondrial genome [25]. These findings support the view that evolutionary loss and simplification have played a major role in shaping the evolution of myxozoans [22, 25, 26].

Here we characterize the genome of *Myxobolus honghuensis*, a parasite of economically important gibel carp [27]. We compare its genome with other myxozoans and free-living Cnidaria to reveal unique and conserved genomic features that characterize this cryptically complex parasite group.

## Results

### Genome assembly and annotation

We sequenced the *M. honghuensis* genome with a PacBio RSII, yielding ~136× coverage and a 16-kilobase (kb) average read length (Additional file 1: Table S1). The final long-read data contained 2,030,357 sequences with a mean length of 10.4 kb (Additional file 1: Table S2). The genome size was estimated by k-mer analysis using Jellyfish software (Additional file 2: Fig. S1) to be 206 megabases (Mb). The FALCON-assembled genome contained 1118 contigs (161 Mb, N50 1.3 Mb; Table 1). *M. honghuensis* has a relatively larger myxozoan genome (Additional file 1: Table S3). The assembly showed high integrity and quality, with >98.5% of Illumina genome survey reads mapped to the PacBio assembly (Additional file 1: Table S4), and successful reconstruction of the nuclear rRNAs. Core Eukaryotic Genes Mapping Approach (CEGMA) [28] identified only 42.7% of CEGs; a low percentage also seen in another myxozoan [25] and possibly due to fast-evolutionary rates rendering even common eukaryotic genes difficult to recognize.

**Table 1:** Comparison of myxozoan genome assembly quality

Species name	*Myxobolus honghuensis*	*Thelohanellus kitauei*	*Myxobolus squamalis*	*Henneguya salminicola*	*Kudoa iwatai*	*Enteromyxum leei*	*Sphaeromyxa zaharoni*
Assembly size (kb)	161,092,274	150,348,159	43,671,844	61,443,780	31,197,353	68,163,509	173,585,031
Number of scaffolds	1118	5757	37,921	18,330	1639	69,053	70,914
Number of contigs	1118	15,632	37,919	18,330	1646	69,178	70,935
Contig N50	1,273,483	13,033	1286	7570	39,532	998	4474
Scaffold N50	1,273,483	149,756	1286	7570	40,195	998	4474
CEGs (complete)	42.7%	46.8%	37.5%	53.6%	73.0%	25.8%	39.1%
%GC	22.8	37.5	27.3	29.0	23.6	33.5	28.0
References	Present study	Yang et al., 2014 [23]	Yahalomi et al., 2020 [25]	Yahalomi et al., 2020 [25]	Chang et al., 2015 [22]	Chang et al., 2015 [22]	Chang et al., 2015 [22]

The *M. honghuensis* genome contains 15,433 inferred protein-coding genes, based on combined ab initio gene prediction, homology searching, and transcript mapping (Table 2, Additional file 1: Table S5). We assigned functions to 39.3% (6072) of the gene models (Additional file 1: Table S6).

**Table 2:** Summary of genome annotation

Genome annotation	
Transposable elements	
LTR	2,838,408 bp
LINE	459,926 bp
SINE	2,326,269 bp
DIRS	339,533 bp
DNA	20,248,630 bp
Total	58,174,337 bp
Protein-coding genes	
Total number	15,433
Mean CDS length	756 bp
Mean exon length	132 bp
Mean intron length	507 bp
Functional annotation	
GO	1371 (8.9%)
KEGG	2918 (18.9%)
KOG	3946 (25.6%)
TrEMBL	4935 (32.0%)
NR	5847 (37.9%)
NT	1826 (11.8%)
Total	6072 (39.3%)

### Transposable element and whole-genome duplication analyses

**Fig. 1:**
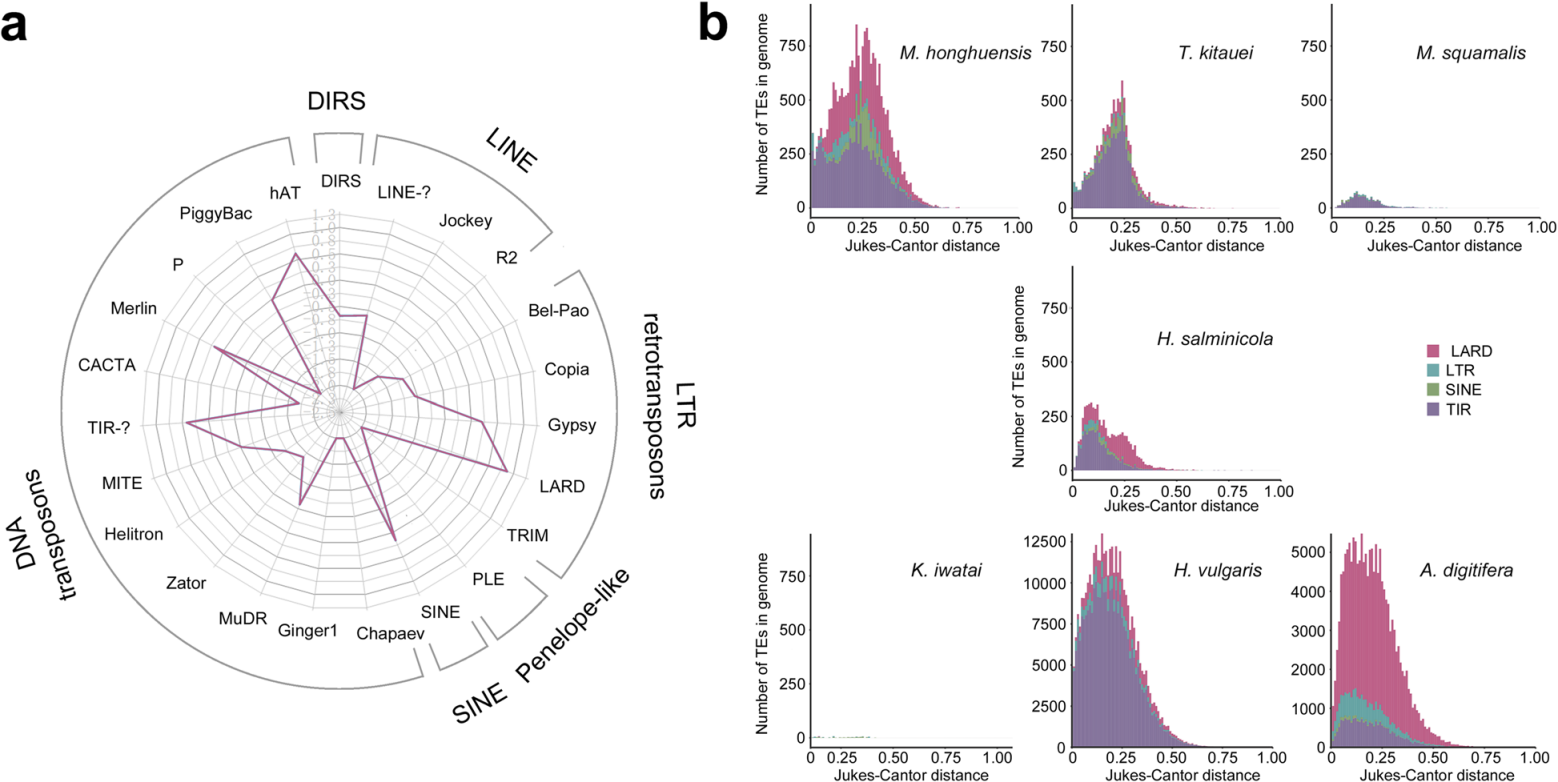
Evolutionary history and expression of transposable elements (TEs). **A** Radar chart showing the proportion of TE superfamilies in the *Myxobolus honghuensis* genome. **B** Copy divergence analysis of TE classes in seven cnidarian genomes, based on Kimura 2 parameter distances. The percentage of TEs in genomes (*y*-axis) are clustered based on their Kimura values. Younger copies are located on the left side while older copies are located on the right side

To identify factors that contribute to the relatively large genome of *M. honghuensis*, we analyzed the transposable element (TE) content and whole-genome duplications (WGDs) in *M. honghuensis* and compared it with other cnidarians [29, 30]. The *M. honghuensis* genome contained 36.1% (55.5 Mb) repetitive sequences, most of which are TEs (23.7% of the total genome; Table 2 and Additional file 1: Table S7). Long terminal repeats (LTRs, 9.2%) and terminal inverted repeats (TIRs, 11.9%) are the major contributors of retrotransposons (11.1%) and DNA transposons (12.6%) respectively (Fig. 1a). We calculated the relative age of transposable element copies using Kimura distance analyses and comparisons with other cnidarians, and revealed that *M. honghuensis* has had at least two transposon bursts (Fig. 1b).

**Fig. 2:**
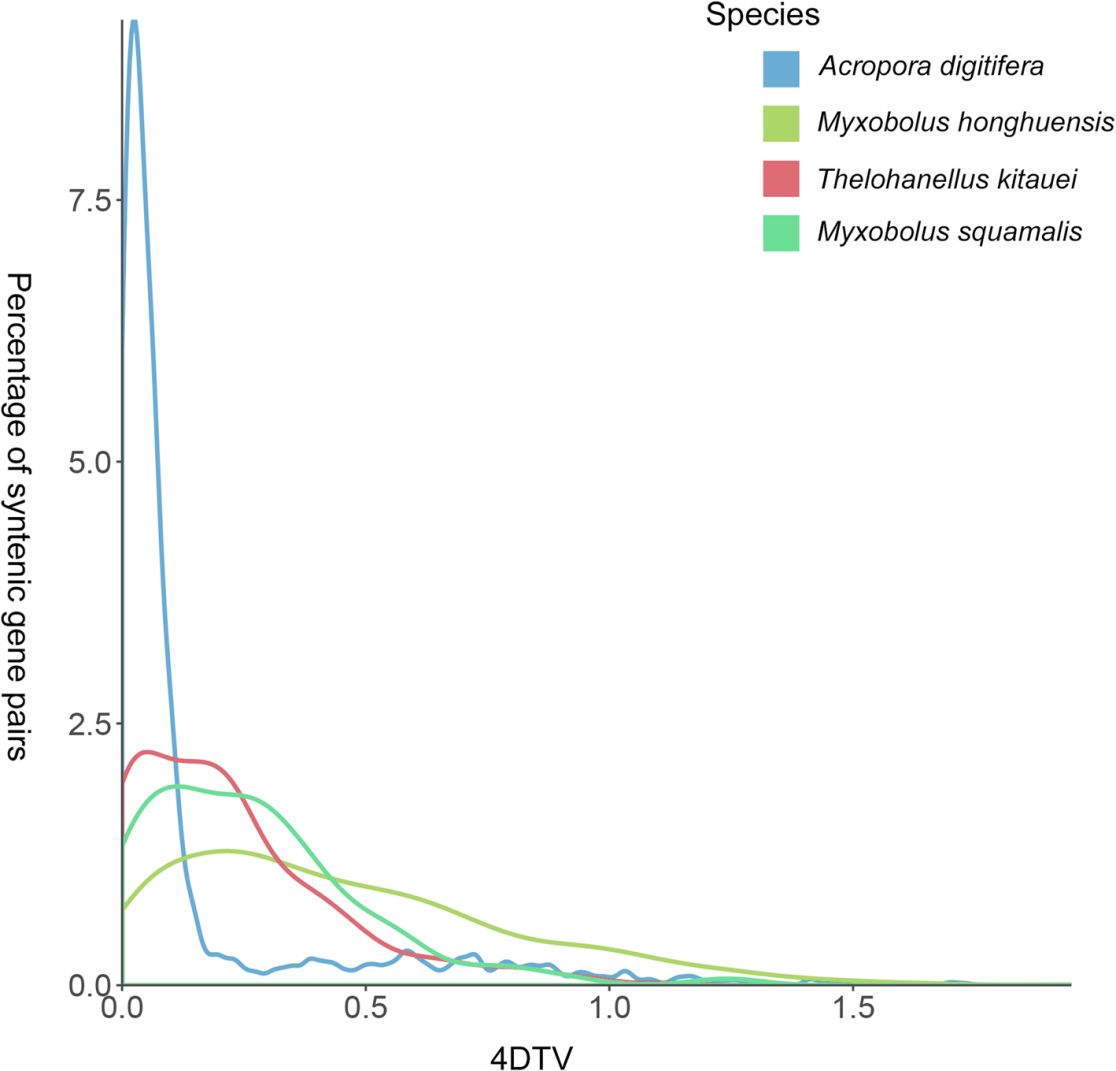
Distribution of the rate of transversions on fourfold degenerate synonymous sites (4DTv) among paralogs in genomes of four cnidarians

To identify potential WGD events in *M. honghuensis*, we determined the number of syntenic gene pairs in five myxozoans and two free-living cnidarian genomes (Additional file 1: Table S8). We found few syntenic blocks and syntenic gene pairs in the *M. honghuensis* genome, suggesting that it has not undergone WGD. We then calculated fourfold synonymous third-codon transversion (4DTv) values (a neutral genetic distance used to estimate the relative timing of evolutionary events [31]) for paralogous gene pairs in the free-living *Acropora digitifera*, and myxozoans *M. honghuensis*, *Thelohanellus kitauei*, and *Myxobolus squamalis*. We observed no sharp peaks in the 4DTv plots of the myxozoans, which supported a hypothesis that WGD has not occurred in these species (Fig. 2).

### Mitochondrial (mt) genome sequences and nuclear-encoded proteins that are targeted to mitochondria

To determine whether there is mt genome in *M. honghuensis*, or it has lost its mt genome, as shown in the case of myxozoan *Henneguya salminicola* [25], we constructed a myxozoan seed database consisted of the complete mt genome of *M. squamalis* (MK087050) and the mt genome-containing sequence (JWZT01002463) of the closely related myxozoan *T. kitauei* [23, 25]. Then, BLASTN and TBLASTX searches were conducted against the PacBio and Illumina genome assemblies using seed sequences (MK087050 + JWZT01002463) as queries. We successfully identified three contigs (scaffold120386, scaffold85385, scaffold38991) in the Illumina genome assembly, which were considered to contain mt genome sequences of *M. honghuensis*. However, there were no positive hits in the PacBio genome assembly (e-value ≤ 1e−25). Then, the three Illumina contigs were added to the original seed to form new seed sequences. BLASTN and TBLASTX searches were further performed using the new seed sequences as queries against the PacBio long-read raw data (e-value ≤ 1e−25). In this way, 298 positive hits were found in the PacBio long-read raw data. These long-read PacBio raw reads were also considered to contain mt genome sequences. Our results showed that while *M. honghuensis* has a mt genome, it could not be recovered successfully using the PacBio assembly tools.

To explore nuclear-encoded protein target to mitochondria in *M. honghuensis*, we built a database of 198 nuclear-encoded proteins that function in mitochondria (e.g., cristae organization, mtDNA replication and translation, electron-transport chains) in fruit fly, human, and *Hydra*, and identified 97 homologs in *M. honghuensis* (Additional file 1: Table S9), 82 in *H. salminicola*, 75 in *Myxobolus cerebralis*, 63 in *M. squamalis*, and 22 in *Kudoa iwatai* [25]. We detected 7/11 cristae organization proteins in *M. honghuensis*, compared with 5/11 in *H. salminicola* and 4–6/11 for the other myxozoans. We identified 33/64 mt electron-transport chain complex proteins in *M. honghuensis*, compared with 10/64 in *H. salminicola* and 18–20/64 in the other published myxozoans. We detected 57/123 genes involved in mt genome replication and translation in *M. honghuensis*, 7/123 in *H. salminicola*, and 41–58/123 in the other myxozoans.

Myxozoans have diverse mitochondrial genome architectures and at least one—*H. salminicola*—has lost its mt genome [25, 32, 33]. We showed that *M. honghuensis* has a fast-evolving mt genome and retains many genes related to aerobic respiration, mitochondrial genome translation and replication, and cristae organization. However, it should be noted that we did not recover any specific complete mt genes of *M. honghuensis* in this study. We assumed that most of our blast hits of mt genome were located in the non-coding areas, which might reside in the large conserved region [25]. In the future work, assembling a complete *M. honghuensis* mt genome from improved long-read sequencing will help us to understand its structure and evolution. Furthermore, DAPI-staining and ultrastructural methods are needed to provide microscopic evidence of the *M. honghuensis* mt.

### Evolution of gene families

**Fig. 3:**
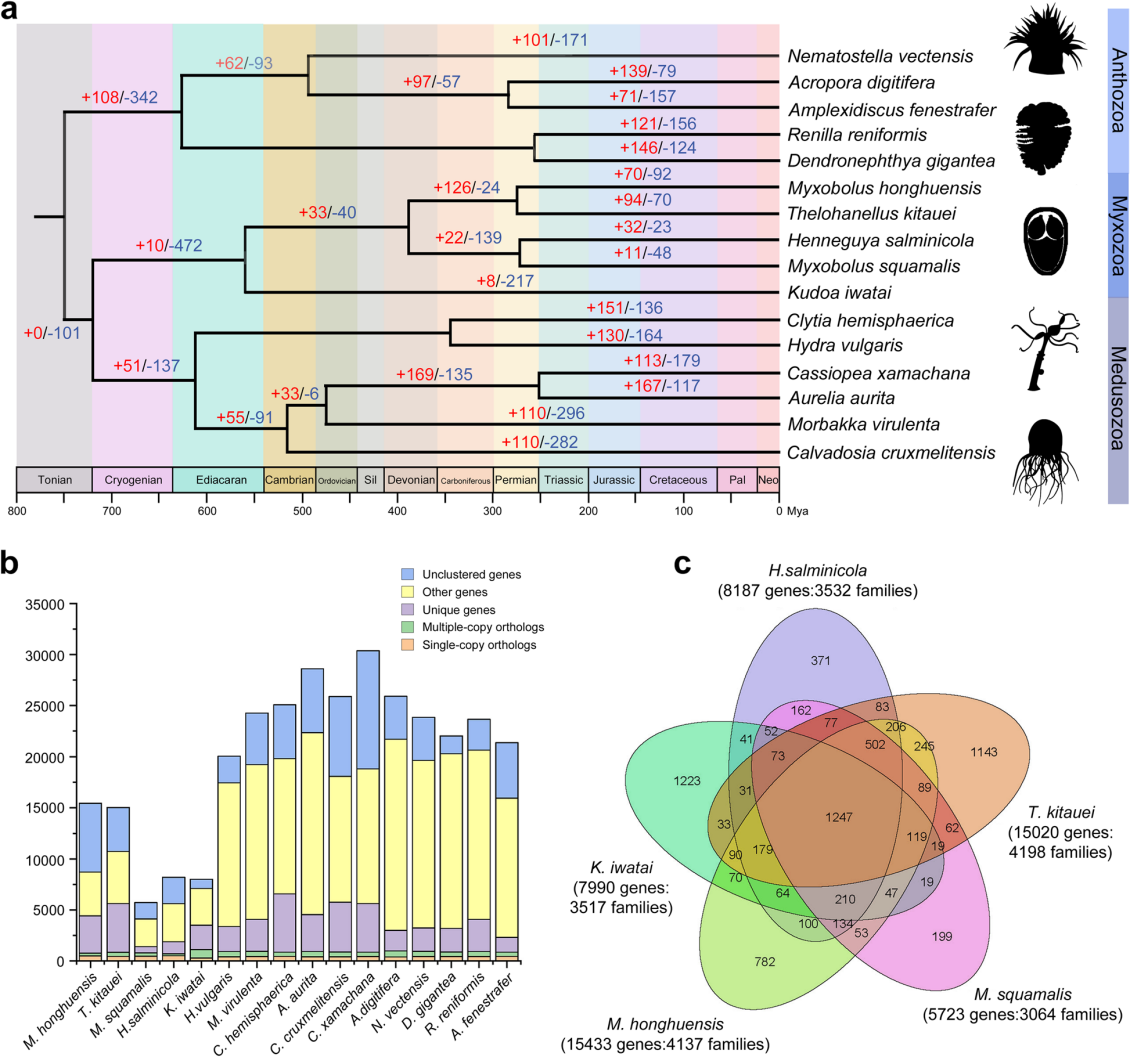
Gene family and phylogenetic analyses of *Myxobolus honghuensis* and other representative cnidarian genomes. Phylogenetic tree was generated from a matrix of 51 single-copy orthologs (11,323 amino acid positions) using maximum likelihood under the GTRGAMMA model. **A** Number of expanded (red) and contracted (blue) gene families in each evolutionary branch. The expanded families are related to energy metabolism, intra- and intercellular communication, and invasion. The contracted families are related to respiratory, neural, and immunity functions, e.g., cytochrome c biogenesis protein, immunoglobulin v-set domain, and clustered mitochondria. **B** Clusters of orthologous and paralogous gene families in *M. honghuensis* and 15 other cnidarians. **C** Overlap of gene families in *M. honghuensis* and four other myxozoans

A comparison of genome sequences by OrthoMCL showed that *M. honghuensis* has 4137 of the 12,300 orthologous gene families identified in 16 cnidarians (Fig. 3b and Additional file 1: Table S10). Compared with other cnidarians, the *M. honghuensis* genome has 10,362 species-specific genes (>67% of the entire gene repertoire) (Additional file 2: Fig. S2, Fig. S3), which were enriched (*p* < 0.05) in single-organism catabolic processes (GO: 0044712), cellular catabolic processes (GO: 0044248), and peptidase activity (GO:0070011) (Additional file 1: Table S11 and Additional file 2: Figs. S4-S6). We identified a core set of 1247 gene families shared among 5 myxozoans (*M. honghuensis*, *T. kitauei*, *H. salminicola*, *M. squamalis*, and *K. iwatai*), which accounted for 29.7–40.7% of their gene families (Fig. 3c). The *M. honghuensis* genome has 70 significantly expanded and 92 significantly contracted gene families based on the *z*-score of gene count differences among 16 cnidarians (Fig. 3a and Additional file 1: Table S12). The expanded families included 2068 genes (Additional file 1: Table S13), which over-represent energy metabolism, intra- and intercellular communication, and invasion (Additional file 2: Fig. S7, Fig. S8). GO enrichment analysis revealed that the expanded families were enriched in meiotic cell cycle processes (GO:1903046), meiotic nuclear division (GO:0007126), and macromolecular complexes (GO:0032991) (Additional file 2: Figs. S9-S11). The 92 contracted families (Additional file 1: Table S14) were related to respiratory, neural, and immunity functions, e.g., cytochrome c biogenesis protein, immunoglobulin v-set domain, and clustered mitochondria (Additional file 2: Fig. S12, Fig. S13). GO enrichment analysis showed that contracted families were enriched in macromolecular complexes, and s-adenosylmethionine-dependent methyltransferase activity (GO:0008757) (Additional file 2: Figs. S14-S16).

### Streamlined neural molecules

**Fig. 4:**
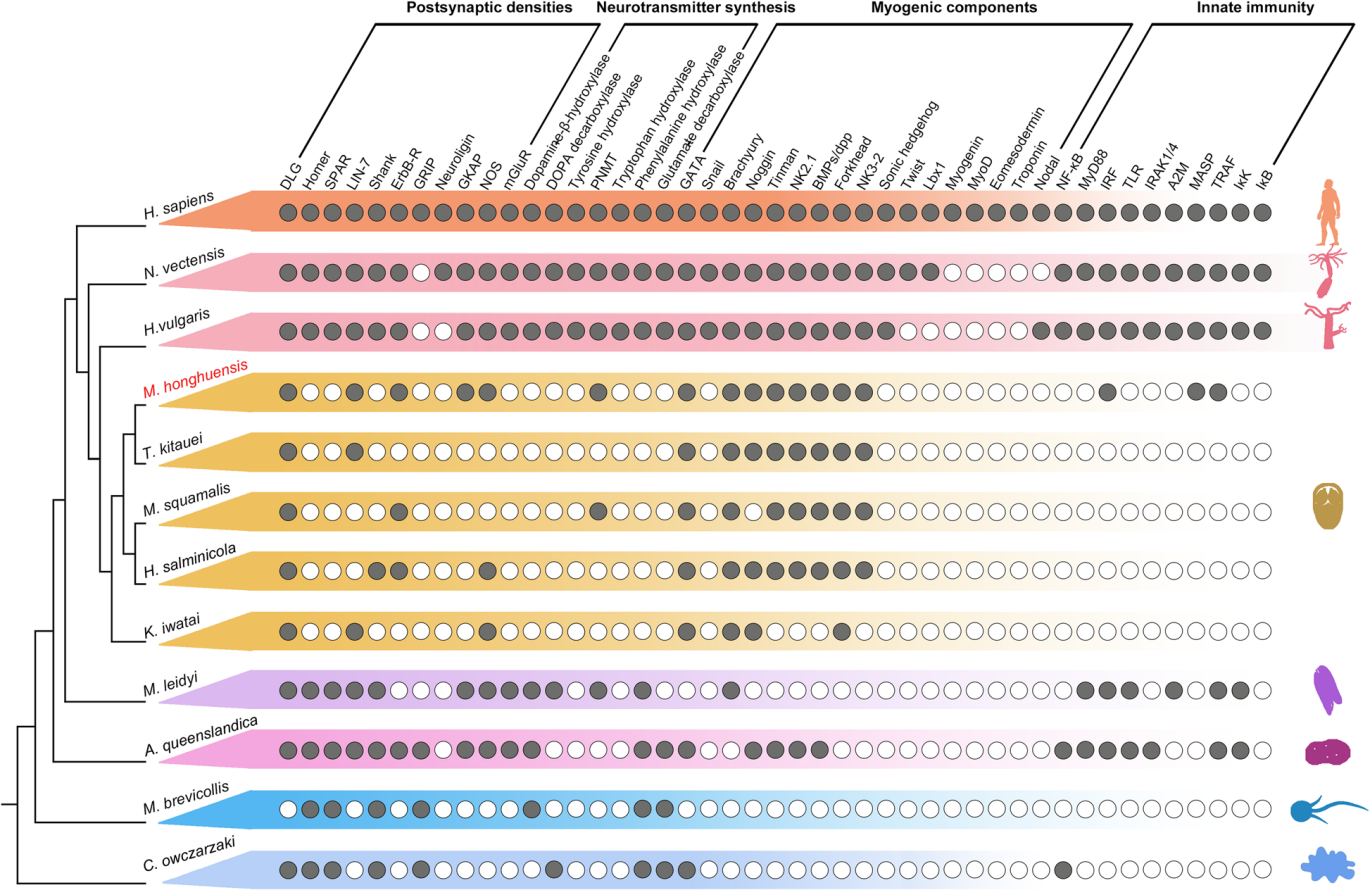
Presence/absence of genes associated with postsynaptic densities (PSD), neurotransmitter synthesis, myogenic components, and innate immunity in myxozoans and other representative eukaryotes

To test if myxozoans retain any components of a nervous system, we searched for genes associated with postsynaptic densities (PSDs) and neurotransmitters (Fig. 4). We found 5/11 PSDs genes (DLG, LIN-7, ErbB-R, GKAP, and NOS) and 1/8 neurotransmitter genes (PNMT), but only in *M. honghuensis* and *M. squamalis* (Fig. 4). Other neural genes found in free-living Cnidaria (Homer, SPAR, Shank, neuroligin, and mGluR) were not detected [34, 35]. We consider the apparent loss of these in *M. honghuensis* to be secondary, except GRIP, which is not present in all cnidarians, and might be lost primarily in myxozoans.

### Loss of innate immune genes

Innate immune components are found in nearly all bilaterians and cnidarians, and recently detected in placozoans, ctenophores, and sponges [36–38]. We found that all 10 major gene families involved in immunity [39] were present in free-living cnidarians, but reduced in the 5 analyzed myxozoans (Fig. 4). The reduced complement of immune system components implies that canonical immune system pathways are not functional in myxozoans.

### Genes that promote the *M. honghuensis* life cycle

*Myxobolus* myxospores can resist drying, freezing, and animal digestion [40]. We identified 235 *M. honghuensis* genes involved in stress resistance (Additional file 1: Table S6), including the universal stress protein Sll1388 and DNA repair and recombination protein RadA, and determined these are under positive selection (Additional file 1: Table S15).

We detected the expansion of genes related to recognition, rapid proliferation, and migration. C-type lectins (CLEC), which recognize complex carbohydrates on cells and tissues [41], are significantly expanded in the *M. honghuensis* genome (Additional file 1: Table S13), as are gene families related to meiotic cell cycle processes and meiotic nuclear division (Additional file 1: Table S16; Additional file 2: Fig. S9). KEGG analysis revealed *M. honghuensis* is enriched in lineage-specific regulators of the actin cytoskeleton (Additional file 1: Table S17), which is involved with cell migration and adhesion [42]. In addition, gene families involved in tumor metastasis and hyperplasia, such as tenascin and extracellular matrix (ECM)-receptor interaction, are significantly expanded (Additional file 1: Table S13), which is in line with the enrichment of *M. honghuensis* lineage-specific genes of cancer pathways (Additional file 1: Table S17). A previous study indicated that the metastasis of cancer cells is partly analogous to the expansion mechanism of protozoan parasites [43]. Since miniaturization has enabled myxozoans to converge on patterns of host exploitation similar to those of protists [19], we suggest that this tumor-related gene expansion, together with enhanced meiotic and actin cytoskeleton activity, played an important role in the migration and rapid proliferation of *M. honghuensis* in host tissues.

Endoparasites live in a nutrition-restricted environment within the host and thus may have unique energy metabolism strategies [44]. We showed that the lineage-specific genes of *M. honghuensis* were enriched in GO terms associated with single-organism and cellular catabolic processes (Additional file 2: Fig. S4). By calculating Ka/Ks ratios, we detected positive selection in genes related to enzymatic catalysis in carbohydrate metabolism (Additional file 1: Table S15). Compared to other cnidarians, we observed marked expansion of gene families involved in fatty acid biosynthesis, elongation, and degradation, (Additional file 1: Table S13), but contraction of low-density lipoprotein receptors (LDLRs) (genes, which reduce cholesterol [45] Additional file 1: Table S14). Reduction of LDLRs might promote accumulation of cholesterol, which could improve success of *M. honghuensis* within its hosts, as has been shown for other parasites including amoeba and flagellates [46, 47].

We observed expansion of gene families related to the cellular processes of nutrient uptake and waste excretion, such as the ATP-binding cassette (ABC)-2 family transporter protein (involved in extra- and intracellular transmembrane nutrition and the extrusion of noxious substances [48]) and the major facilitator superfamily (MFS) facilitates the movement of small solutes across cell membranes and is responsible for drug metabolism and metabolite transport in other parasites [49, 50]) (Additional file 1: Table S13). Endocytosis pathways were also expanded (Additional file 1: Table S13). We observed genes linked with probable enhancement of intra- and intercellular communications in the *M. honghuensis* genome; e.g., the glycosphingolipid biosynthesis pathway, which mediates and modulates intercellular coordination [51] (Additional file 1: Table S18). Also, we found significant expansion of genes encoding Notch ligands Delta and Serrate and the pathway involved with gap junction, which play key roles in intercellular communication [52, 53] (Additional file 1: Table S13). Relative to other cnidarians, *M. honghuensis* has more genes of the focal adhesion pathway, which regulates communication between the cell and the surrounding extracellular matrix [54] (Additional file 1: Table S13).

### Mesoderm and myogenic components

The mesoderm is a germ layer present in triploblastic animals that gives rise to skeletal structures, circulatory organs, and muscle tissue. However, both genes associated with mesoderm and muscle formation, and mesoderm-like musculature have been found in cnidarians [55]. In the Myxozoa, muscles and mesodermal musculature are only known from Malacosporea with worm-like life stages, not Myxosporea (which includes *M. honghuensis*) [56]. Here, as expected from prior work [57, 58], we did not detect myogenin, MyoD, eomesodermin, and troponin in any myxozoan or free-living species (Fig. 4), genes that typically play a role in muscle differentiation, specification, contraction, and atrophy [59]. We did however identify GATA, Brachyury, and Forkhead in the free-living cnidarians and myxozoans. These conservative transcription factors are involved in specification and differentiation of multiple cell types during embryogenesis and development [60, 61]. We did not detect either of two developmental regulator genes, sonic hedgehog and SNAIL, in the myxozoans, but found them in the free-living species as expected [62, 63] (Fig. 4).

### Wnt, Hedgehog, and Homeobox

**Fig. 5:**
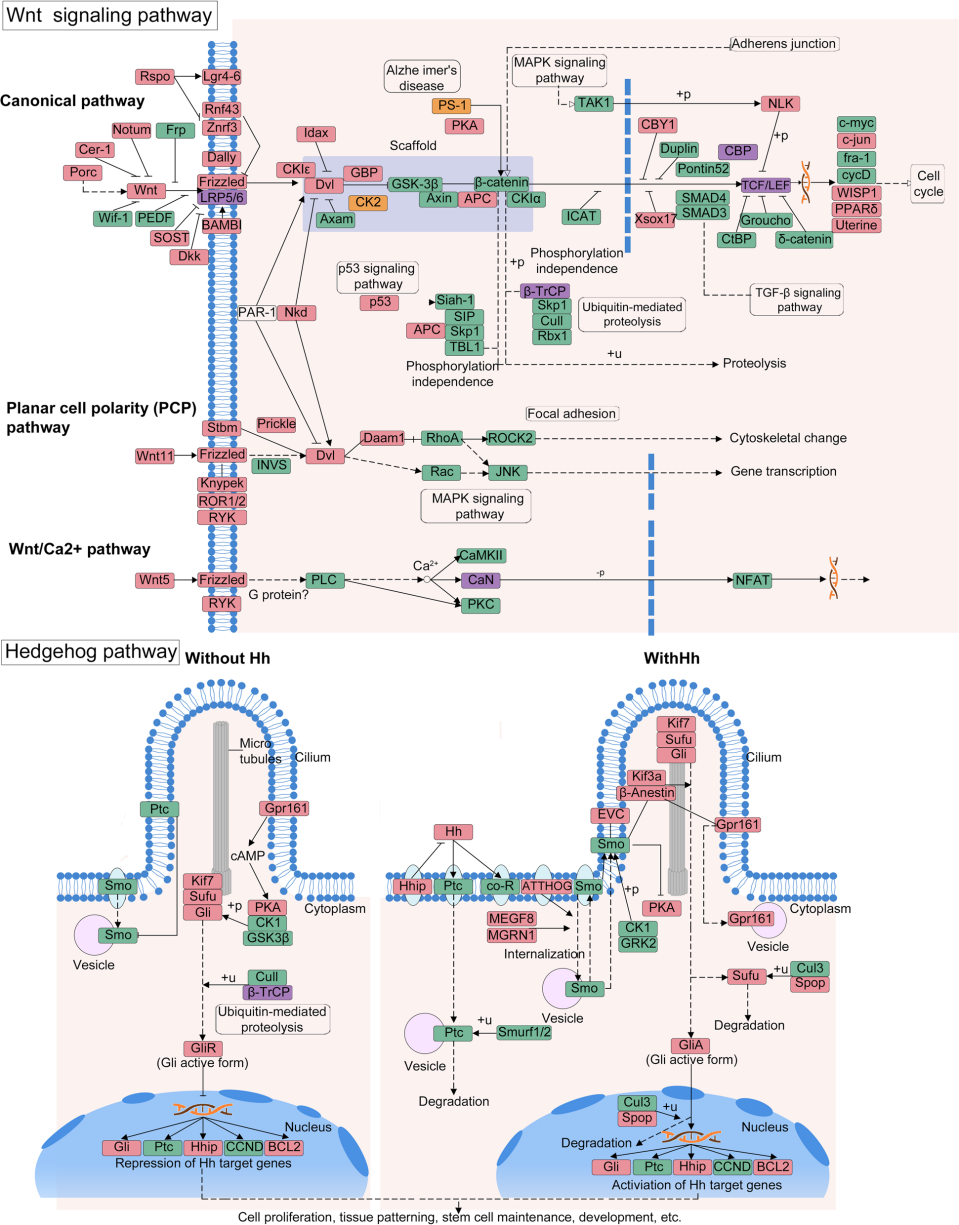
*Myxobolus honghuensis* genes involved in the Wnt and Hedgehog pathways. Components present in the *M. honghuensis* genome are in green, undetected components are in red. The purple and orange boxes indicate expanded and contracted genes respectively. Abbreviations, annotations, and connections are adapted from KEGG pathway (04310 and 04340) and follow KEGG standards

The diverse life cycle development and body forms of cnidarians are regulated in part by homeobox genes, and the Wnt and Hedgehog signaling pathways, which determine body polarity, tissue identity, and polyp-to-jellyfish transition [64, 65]. In *M. honghuensis*, we detected 43/83 Wnt signaling pathway genes, including 9 key genes (Fig. 5) that could control neuronal development, left-right axis establishment, and mesoderm segmentation. Ten genes in the Wnt pathway are expanded (Additional file 1: Table S13), including LRP5 and beta-TrCP, which trigger beta-catenin signaling and mediate ubiquitination, respectively [66]. Two genes that stabilize beta-catenin and regulate many cellular processes, PS1 and csnk2b [67], are contracted (Additional file 1: Table S14). We detected 21/52 components of the Hedgehog pathway, including growth arrest-specific 1 (Fig. 5). More Wnt and Hedgehog signaling pathway genes were found in the *M. honghuensis* genome (43/83 = 51.8%, 21/52 = 40.4%) than in the other myxozoans *K. iwatai* (22/72 = 30.6%, 4/19 = 21.1%) and *M. cerebralis* (23/72 = 31.9%, 5/19 = 26.3%). We found 7/24 of ANTP class Homeobox genes in *M. honghuensis* (1 Hox-like and 6 NK-like), compared to 18-24/24 in free-living Cnidaria (Additional file 1: Table S19).

## Discussion

While it seems intuitive that the evolutionary history of obligate parasites is characterized by reductions, including a massive loss of genetic and functional diversity [7], herein we show that the genome of the myxozoan *M. honghuensis* illustrates both reduction and expansion of different components—a pattern described as *mosaic evolution*. Comparison of this parasite with other myxozoans and free-living Cnidaria revealed aspects of genome size variation, adaptive mechanisms, and genome organization, which expand our understanding of parasite genome complexity and evolution.

### Genome size and transposable elements

The genome size of *M. honghuensis* is 206 Mb, with a smaller final assembly size of 161 Mb likely due to abundant repetitive sequences. These estimates place *M. honghuensis* as having a slightly larger genome than closely related *T. kitauei* (188.5 Mb) [23], ninefold larger than the smallest (*K. iwatai*, 22.5 Mb) [22]. The *M. honghuensis* genome is equivalent in size to some free-living cnidarians, including *Nemopilema nomurai* (213 Mb) [68] and *S. malayensis* (185 Mb) (Additional file 1: Table S3) [69], despite having a body size at the micron scale, compared with vastly larger *N. nomurai* (up to 2 m in diameter) [70] and *S. malayensis* (up to 13.5 cm in diameter) [71]. Concomitant with its larger genome size, *M. honghuensis* has 2.8 times the number of protein-coding genes than the distantly related myxozoan *K. iwatai* (5533 genes, 22.5 Mb) [22], but is comparable to that of the more closely related *T. kitauei* (16,638 genes, 188.5 Mb) [23] (Additional file 2: Fig. S17), and similar to free-living cnidarians *Hydra vulgaris* (16,839 genes, 1,005 Mb) [72], and *Nematostella vectensis* (18,000 genes, 450 Mb) [73] (Additional file 1: Table S3). The *M. honghuensis* genome is less compact (mean intron size 507 bp) compared with myxozoans *K. iwatai* (82 bp), *T. kitauei* (240 bp), and the non-myxozoan *Sanderia malayensis* (381bp; Additional file 1: Table S3). *M. honghuensis* has a mean exon size of 132 bp, compared with 102 bp in *K. iwatai*, 235 bp in *T. kitauei*, 218 bp in *H. vulgaris*, and 208 bp in *N. vectensis* (Additional file 1: Table S3).

Transposable element (TE) and whole-genome duplications (WGD) contribute to genome size variation [29, 30]. We found that the *M. honghuensis* genome differs from that of most animals by possessing more DNA transposons than retrotransposons, and no evidence of WGD. We did not detect evidence for WGD in other myxozoans, but confirmed a likely ancient genome duplication in the free-living cnidarian *A. digitifera*, previously reported by Mao and Satoh, 2019 [74]. TE abundances in the myxozoans we analyzed were proportional to their genome sizes, which suggested that differences in myxozoan genome sizes are caused partly by different TE insertion/expansion rates. Massive mobilization of TEs has been implicated in many cases of adaptive evolution, such as adaptation to novel environments, stressors, or environmental change [75], and is thus a likely feature of many myxozoans given their extensive colonization of aquatic and even terrestrial hosts and habitats [76]. A further genetic signature of a dynamic evolutionary history related to parasitism was the finding that *M. honghuensis* transposons were acquired in two bursts, which we propose is an artifact of the host-acquisition or host-switch events that likely occurred several times in evolution of the Myxozoa [77]. The TE results, together with our findings of gene number and intron size, suggest that gene retention, large intron, transposon insertion, but not polyploidy, are the major factors contributing to the relatively large genome size of *M. honghuensis*.

### Species-specific adaptations

*M. honghuensis* has 10,362 species-specific genes (>67% of its gene complement), compared with 1.4 to 63.6% for other animals [78–80]. This high proportion could be integral to adaptations for parasitism in alternate fish and worm hosts. We also found that although *M. honghuensis* has about the same number of expanded (70) versus contracted (92) gene families, many more genes are represented in the expanded families (2068) than contracted (only 90). This suggests that there have been large-scale duplication events leading to expansion of genic content in some families. Myxozoans must succeed in diverse environments during their 2-host life cycles, including invasion and development within multiple host tissues, and persistence in the external environment as spores. We found expansion and positive selection of a series of genes conceivably related to this parasite life cycle, including temperature and salinity stress resistance (Sll1388), and DNA repair (RadA). The strong positive selection of these stress resistance proteins may help spores survive in both the water column, and within the digestive system of their target hosts.

Host invasion by a myxozoan involves molecular and physical evasion of immune responses. We found genes coding for C-type lectins (CLEC) were expanded in *M. honghuensis*, and thus may be an important tool for mediating immunological recognition within its hosts, much as other parasites release CLEC homologous to key host receptors to possibly interfere with immune response or effector function [81]. Myxozoans require motile stages both to avoid host immune responses [82] and to reach target tissues. In *Ceratonova shasta* and *Ceratomyxa puntazzi*, actin has an important role in stage motility [83, 84], and our finding that here *M. honghuensis* is enriched in lineage-specific regulators of the actin cytoskeleton (Additional file 1: Table S17), suggests that actin may be important for success of diverse myxozoan taxa.

### Primitive nervous system?

While free-living cnidarians have simple nerve nets [85], there is no evidence that similar nerves are present in myxozoans even though they can sense proximity of hosts and can have motile, multi-cellular stages [56]. In *M. honghuensis*, we detected 5 genes associated with postsynaptic densities, which raises the possibility that *M. honghuensis* have a miniaturized nervous system, perhaps similar to those typical of insects, especially parasitoids [86]. Both parasitoids and myxozoans have undergone extreme reduction in size during their evolutionary history, with insects retaining a nervous system [87, 88]. Retention of a genetic signature of nervous systems supports the idea that general evolutionary simplification of morphology should not be assumed to have corresponding losses in the genome [34]. In the future work, we can investigate the nerve-like structures in different myxozoans by immunogold labeling of synaptic proteins [89] or immunohistochemical labeling of FMRF-amide and α-tubulin [90].

### Wnt, Hedgehog, and Homeobox

Myxozoans have diverse morphologies in their life cycles, which involve bilateral myxospores, triradial actinospores, and amoeboid multi-nucleate/-cellular sporoplasms. Little is known about the genetic basis of myxozoan development and body patternation, but at least two species have lost the ligands, receptors, and most downstream elements of the Wnt and Hedgehog signaling pathways [22], and no pathway-specific components were detected in a recent in-depth analysis of *Tetracapsuloides bryosalmonae* from both of its hosts [91]. In contrast, we identified Wnt and hedgehog components in *M. honghuensis*, demonstrating that at least *M. honghuensis* retain these signaling components (e.g., LRP5 and beta-TrCP) while losing others (e.g., PS1 and csnk2b). However, it should be noted that most of the ligand and/or receptor are still missing in the Wnt and Hedgehog pathways, and it is likely that the other elements are non-functional or operating in other pathways. For the homeobox, *M. honghuensis* is only the second myxozoan (after *T. bryosalmonae* [91]) in which unambiguous Homeobox-family genes/proteins have been observed. The identification of homeobox will facilitate future functional studies and spatial expression mapping to better understand whether and how these genes are involved in the radical morphological shifts that occur between myxozoan life stages (spores versus amoeboid sporoplasm versus plasmodia). Particularly informative will be discovery of how homeobox genes control the bilateral/triradial alternation between actinospore and myxospore stages, which should be tractable in the few species whose complex life cycles are both known and maintainable in the laboratory (*C. shasta* [92], *Parvicapsula minibicornis* [93], *M. cerebralis* [94]).

### High genomic variation across Myxozoa

We revealed high genomic variation within myxozoans, including a wide range of genome sizes across myxozoans and a high number of non-overlapping gene families between species (Fig. 3c). Even between two closely related species, *M. honghuensis* and *T. kitauei*, the species-specific components account for a large proportion of the genome and several gene families, such as PSDs, neurotransmitter synthesis, and innate immunity, exhibit clear different pattern of presence and absence. This phenomenon raises the questions of why there is so much variation across myxozoa and why some species can survive without these gene pathways being retained and expanded. In fact, the variation in genome size and content has also been reported in other parasites, such as microsporidians [95], mesozoans [9, 16], and parasitic nematodes [10]. It is widely appreciated that parasites are prone to rapid evolution, and because of their often short generation times, large population sizes, and host-parasite arms races, parasite genome may show evidence of adaptive “plasticity” [4, 96]. Therefore, a possible answer for the above questions is that myxozoans exhibit highly species-specific adaptations to variable environments (e.g., different habitats, hosts, organs, and tissues), which are shown to be related to substantial genome size variation and increased proportion of non-overlapping gene families [80, 97, 98]. However, the causes of myxozoan genomic variation are still widely open for discussion and more works needs to be done to investigate the genomes from a diverse range of myxozoans.

## Conclusions

In summary, we show that *M. honghuensis* has a relatively larger myxozoan genome, which is both less reduced and less compact, due to gene retention, larger introns, and transposon insertions, but not polyploidy. As evolutionary adaptations to endoparasitism, the *M. honghuensis* genome evolved in a mosaic fashion of conserved, divergent, depleted, and enhanced genes and pathways. It has the simplest animal immune components and has retained a reduced set of neurological and signaling genes, including components of the Wnt and Hedgehog pathways, and Homeobox domains. *M. honghuensis* has increased the number of genes associated with stress resistance, tissue invasion, energy metabolism, and cellular processes. These results illustrate that *M. honghuensis* genome evolution is not governed solely by streamlining, but rather embodies trade-offs between genomic simplification and complexification necessary for success of a multi-host parasite life cycle. Altogether, this study changes our view of parasite evolution, and furthermore provides an exciting new system and genomic resources to investigate the evolutionary plasticity and function of core cellular mechanisms in animals.

## Methods

### Sampling, DNA/RNA extraction and sequencing

*M. honghuensis* was collected from infected allogynogenetic gibel carp *Carassius auratus gibelio* in Zoumaling Farm, Hubei Province, China on July 29, 2015. Fish (66) were held on ice before being killed with an overdose of MS-222 (Sigma-Aldrich, Co., Ltd., St. Louis, MO, USA). From each fish, tissue containing one large cyst was homogenized by a manual glass tissue grinder and suspended in 0.1M phosphate-buffered saline (PBS), pH 7.2, then filtered through cotton gauze. Myxospores were separated from the filtrate by sucrose gradient centrifugation and Percoll gradient centrifugation in turn, washed several times with distilled water, and then examined microscopically to verify purity and identify. Purified myxospores were placed into RNAlater (Sigma) or 95% ethanol, frozen in liquid nitrogen and finally stored at – 80 °C. Samples with highest myxospore number and purity were selected for downstream processing: 15 for DNA, and 3 samples for RNA sequencing. All of these 18 samples were identified as *M. honghuensis* based on morphology and 18S sequencing [99, 100] (Additional file 1: Table S20). The maintenance and care of experimental animals complied with the National Institutes of Health Guide for the care and use of laboratory animals [101] and was approved by the animal care and use committee of Huazhong Agricultural University, China.

From the 15 DNA samples (from 15 different fishes), 3 of them were sent for Illumina genome survey. Genomic DNA was isolated and extracted using the CTAB method [102]. Libraries were built by TruSeq DNA library kit (Illumina). The ~500 bp insert size libraries were sequenced on Illumina HiSeq 2500 (2 × 125 PE). The 3 DNA candidates were also sent for PacBio library construction. To do this, 10 μg gDNA was sheared to an ~20-kb targeted size using a Covaris g-Tube, then used for size selection using Blue Pippin (Sage Science, Beverly, MA, USA). Then, 20-kb PacBio libraries were prepared following the standard PacBio protocol. After some trial and error (considering the DNA amount, contamination, library quality), only one sample (No. 51, it is unmixed) was sent for final PacBio sequencing. It was sequenced on a PacBio RSII platform (PacBio Sequel sequencer at Biomarker Technologies Corporation) with P6-C4 chemistry.

We have 3 RNA candidates (from three different fishes) for Illumina transcriptome sequencing. Before RNA extraction, we checked the three samples and found immature plasmodia that contained spores of different stages. Then we extracted the RNA separately, tested the RNA quality, and combined the RNA. RNA was isolated with TRIzol 550 (Invitrogen). The purity and integrity of RNA were assessed using NanoPhotometer® spectrophotometer (IMPLEN, CA, USA) and RNA Nano 6000 Assay Kit of the Agilent Bioanalyzer 2100 system (Agilent Technologies, CA, USA) respectively. Only one library was constructed from the combined RNA and sent for RNA sequencing. Libraries were prepared using NEBNext® Ultra RNA kit (New England Biolab (NEB), Ipswich, MA) following the manufacturer’s recommendations and sequenced as 2 × 125 paired-end (PE) runs with Illumina HiSeq 2500. Raw sequence data from both the Illumina genome and transcriptome sequencing were cleaned and trimming of low-quality reads and adaptors using Trimmomatic v0.33 [103]

### 17-mer analysis and evaluation of genome size

All the filtered Illumina paired-end genomic reads were used for 17-mer frequency analysis. Jellyfish v2.1.3 [104] was used to count *K*-mer occurrences with a setting of *K* = 17, and a histogram of *K*-mer distributions was generated. Peak coverage was taken to be the average *K*-mer coverage, and genome size estimated by the formula: G = *K*-mer_num/Peak_depth, where the *K*-mer_num and Peak_depth are the total number and the average depth of 17-mer, respectively.

### Genome assembly and decontamination

We assembled PacBio long-read data of *M. honghuensis* using FALCON v0.7 [105], then used arrow v2.2.2 [106] to polish this long-read-based genome through PacBio reads themselves. Next, we used two rounds of Pilon-based v1.22 [107] polishing to correct single-base variants, indels and to fill the gaps in the final assembly using Illumina genome survey raw data. To evaluate and remove contamination from genome, we first mapped the raw Illumina genome sequences to the common carp genome (NCBI: GCF_000951615.1, [108]) using SOAP software v2.21 [109]. This resulted a very low total mapped rate of 0.02% (Additional file 1: Table S21). This means that there is very few host contamination in our DNA. The Illumina sample is the same as that was used to make DNA for Pacbio. So for the Pacbio assembly, instead of searching against a host genome directly, we applied a customized decontamination protocol in our recent CCPRD framework [110], which consider both host and bacterial contamination. Briefly, Blobtools v1.0 [111] was used to construct a BlobDB, which contained the genome sequences, coverage, and species information. The results were processed by the Blobtools function “create” to annotate each scaffold. A TAGC was drawn at the rank of the phylum and under the taxrule “bestsum.” Using the Blobtools function “view,” taxonomically annotated non-cnidarian scaffolds with a bit-score of ≥200 were inspected manually and compared against the NCBI nucleotide database (BLASTN, -e value 1 × 10 652 −5, -max_target_seqs 20, -outfmt 6). Sequences strongly matched to Chordata and Proteobacteria were excluded. The bacterial sequences were extracted and independently assembled using wtdbg (https://github.com/ruanjue/wtdbg) (Additional file 1: Table S22). CEGMA v2.5 [28] was used to assess the completeness of the genome by mapping against genes that are highly conserved in eukaryotes.

### Annotation of repeats

We used both homology-based and de novo approaches for repeat annotation. Using default settings, LTR_FINDER v1.0.5 [112], MITE-Hunter v1 [113], RepeatScout v1.0.5 [114], PILER-DF v1 [115] were used to build a de novo repeat library from our assembly. The predicted repeats were then classified using PASTEClassifier v1 [116] and merged with Repbase v19.06 [117]. Then, consensus sequences from the repeat library were used as queries for RepeatMasker v4.0.5 [118] to determine the repeat content of the *M. honghuensis* genome. The relative age of the different transposable element (TE) families was estimated through Copy Divergence Analysis (CDA) using Jukes-Cantor distances [119] between individual copies and their consensus sequence. The percentage differences between identified TE copies in the genome and the consensus sequences in the TE library were extracted from the RepeatMasker file (.out file), and converted to Jukes-Cantor distance by the formula *d* = −(3/4)log_e_(1−(4/3)*p*).

### Gene prediction

We used ab initio prediction, homology search, and transcriptome-assisted prediction, for gene prediction in the repeat-masked genome. For the ab initio prediction, we used Genscan v2.1 [120], Augustus v3.2.3 [121], GlimmerHMM v3.0.1 [122], GeneID v1.4.4 [123], SNAP v2013-02-16 [124], with default parameters. For homology prediction, we used GeMoMa v1.3.2 [125] with the protein sequences from *T. kitauei* (NCBI: GCA_000827895.1, [23]), *A. digitifera* (NCBI: GCF_000222465.1, [126]), *Exaiptasia pallida* (NCBI: GCF_001417965.1, [127]), *H. vulgaris* (NCBI: GCF_000004095.1, [72]), *N. vectensis* (NCBI: GCF_000209225.1, [73]), *Orbicella faveolata* (NCBI: GCF_002042975.1, [128]), *Caenorhabditis elegans* (NCBI: GCA_000002985.3, [129]). For transcriptome-assisted gene prediction, we used Hisat2 v2.1.0 and Stringtie v1.3.3 [130] for the transcriptome assembly, then TransDecoder v5.3.0 [131] and GeneMarkS-T v5.1 [132] for gene prediction, and PASA v2.3.3 [133] to predict unigenes from de novo transcriptome assembly. To integrate data derived from these three methods into an EVM-derived gene set, we used EvidenceModeler (EVM) v1.1.1 [134]. Sequences of predicted genes were searched against GO, KEGG, KOG, Swissprot, TrEMBL, NR, and NT databases for annotation.

To annotate non-coding RNAs, we used tRNAscan-SE v2.0 [135] software to predict the tRNAs with eukaryotic parameters. Non-coding RNAs (miRNAs and rRNAs) were detected using BLASTN to search the Rfam [136] database.

### Identification of mt genome sequences and nuclear-encoded proteins that are targeted to mitochondria

To detect whether there is mt genome in *M. honghuensis*, we searched the mt genome sequences in *M. honghuensis* Pacbio contigs, Illumina contigs (from genome survey), and PacBio long-read raw data respectively. First, we constructed myxozoan seed sequences containing mt genome sequences of the closely related myxozoan *T. kitauei* (JWZT01002463) and complete mt genome of *M. squamalis* (MK087050). Then, BLASTN and TBLASTX searches were conducted against the Pacbio and Illumina genomes using seed sequences (MK087050 + JWZT01002463) as queries. The positive hits (e-value ≤ 1e−25) were obtained and added to the seed sequences. Second, BLASTN and TBLASTX searches were performed using the new seed sequences as queries against the Pacbio long-read raw data (e-value were−25).

To search for presence and absence of nuclear-encoded proteins target to mitochondria in *M. honghuensis*, we first built a database based on Yahalomi et al [25], which contains 198 selected nuclear-encoded proteins that function in mitochondria from fruit fly, human, and/or Hydra proteome. These proteins include (i) proteins involved in cristae organization; (ii) genes involved in mitochondrial respiratory chain complexes (I-V); (iii) nuclear genes involved in the replication and translation of the mt genome. Using reciprocal BLASTP, the presence/absence of these proteins were determined in *M. honghuensis* proteome. The *M. honghuensis* proteome includes PacBio gene models and CCPRD from our previous work [110]. The 198 nuclear-encoded proteins target to mitochondria were used as seeds to search candidate homologous proteins in the *M. honghuensis* proteome (e-value to s−3). The candidate proteins were BLASTed against the UniProtKB/Swiss-Prot. Only those proteins with the best match to the same term of seed sequences were considered as present.

### Gene family and phylogenetic analysis

The protein data of representative cnidarian species, including *T. kitauei* (NCBI: GCA_000827895.1, [23]), *K. iwatai* (NCBI: GCA_001407235.2, [22]), *H. salminicola* (NCBI: GCA_009887335.1, [25]), *M. squamalis* (NCBI: GCA_010108815.1, [25]), *H. vulgaris* (NCBI: GCF_000004095.1, [72]), *Morbakka virulenta* (NCBI: GCA_003991215.1, [65]), *Clytia hemisphaerica* (NCBI: GCA_902728285.1, [137]), *Aurelia aurita* (NCBI: GCA_004194415.1, [65]), *Calvadosia cruxmelitensis* (NCBI: GCA_900245855.1, [138]), *Cassiopea xamachana* (NCBI: GCA_900291935.1, [138]), *A. digitifera* (NCBI: GCF_000222465.1, [126]), *N. vectensis* (NCBI: GCF_000209225.1, [73]), *Dendronephthya gigantea* (NCBI: GCA_004324835.1, [139]), *Renilla reniformis* (NCBI: GCA_900177555.1, [140]), and *Amplexidiscus fenestrafer* (NCBI: PRJNA354436, [141]), were used for gene family clustering (Additional file 1: Table S23). The 16 cnidarians were chosen because they have relatively good genome quality and gene models. So, we can get more accurate estimation of gene family expansion and contraction. The gene family, phylogenetic and molecular clock analyses need to be conducted on the same tree. So the latter two analyses also used these 16 species. We used OrthoMCL v2.0.5 [142] to cluster orthologous groups among selected species, with low-quality gene model sequences removed based on the sequence length of OrthoMCL criteria. Sequences were used for all-versus-all BLAST searches with e-value cutoff of 1e−5. Homologous gene families of various species were determined using OrthoMCL with the Markov model. The Venn diagram of shared orthologous groups between myxozoans *M. honghuensis*, *T. kitauei*, *K. iwatai*, *H. salminicola*, and *M. squamalis* was plotted using R.

Fifty-one single-copy orthologous gene clusters of these 16 Cnidaria species were extracted from OrthoMCL results, and aligned using MAFFT v7.205 [143], poorly aligned regions were eliminated by GBlocks v0.91b with default parameters and trimmed sequences were concatenated to form a supermatrix of 16 taxa and 11,323 amino acid positions for phylogenetic analyses. We constructed a phylogenetic tree using RAxML v8.2.12 [144] with the GTRGAMMA model and 100 rapid bootstrap replicates.

### Molecular clock and CAFÉ analysis

We estimated species divergence times through the Bayesian relaxed molecular clock approach using MCMCtree in PAML v4.8 [145]. Fossil records were downloaded from the TIMETREE [146] website for calibration. Three calibration points were used: the maximum age of Medusozoa + Myxozoa were set to and 680 million years ago (Mya), the node of crown cnidarians was constrained with a minimum age of 677 Mya and maximum age of 805 Mya, the minimum and maximum divergence age of sea anemones and stony corals were set to 437 Mya and 600 Mya. Expansion and contraction of gene clusters were determined using CAFÉ v4.1 [147]. The phylogenetic tree and the divergence times from the previous step were used in CAFÉ to infer changes in gene family size using a probabilistic model.

### Detection of positively selected genes

To detect the positively selected genes in *M. honghuensis*, the single-copy genes of *M. honghuensis* and from the closely related species *K. iwatai* were aligned using MAFFT. The estimation of dN and dS values were obtained using yn00 software (part of the PAML program package). Genes with a dN/dS ratio >1 were considered as indicative of gene parts displaying a signal of positive selection. The positively selected genes were annotated by GO, KEGG, KOG, Pfam, Swissprot, and NR analyses as for the entire genome.

### Gene gain and loss

Using reciprocal BLASTP, the presence/absence of the conserved developmental signaling pathways (Wnt and Hedgehog), neuronal signaling, myogenic components, and innate immune regulation were determined in five myxozoans (*M. honghuensis*, *T. kitauei*, *K. iwatai*, *H. salminicola*, *M. squamalis*). Briefly, *Homo sapiens* proteins (NCBI: GCA_000001405.28, [148]) were used as seeds to perform reciprocal BLAST searches against the proteome of the two free-living cnidarians, *H. vulgaris* and *N. vectensis*. Then the *H. sapiens*, *H. vulgaris*, and *N. vectensis* proteins were used to identify candidate homologous proteins in myxozoan predicted proteomes (e-value ≤ 1e−3). The candidate proteins were BLASTed against the UniProtKB/Swiss-Prot. Only those proteins with the best match to the same term of seed sequences were considered as present. As a control of our approach, we confirmed presence of these genes in ctenophore *Mnemiopsis leidyi* (NCBI: GCA_000226015.1, [149]), sponge *Amphimedon queenslandica* (NCBI: GCA_000090795.1, [39]), and two unicellular eukaryote species, *Monosiga brevicollis* (NCBI: GCA_000002865.1, [150]) and *Capsaspora owczarzaki* (NCBI: GCA_000151315.2, [151]). Additionally, using the same reciprocal BLAST searches and human and fruit fly proteins as seeds, we searched the 20 meiosis-related genes that were conserved among human, fruit fly, budding yeast, and dinoflagellates [152] in the genome and transcriptome of *M. honghuensis*.

### 4DTv analysis

To detect any whole genome duplication events in myxozoan genomes, we searched for putative paralogs within genomes using MCscanX v2.49 [153] and calculated transversion rates of fourfold generation sites (4DTv) between gene pairs located in synteny blocks using an in-house Perl script (https://github.com/qingxiangguo/scripts). For comparison and as a control, we analyzed protein sequences of *A. digitifera*, a cnidarian known to have experienced genome duplication [74]. For the initial 4DTv analysis, we have done syntenic gene analysis for 7 species in Additional file 1: Table S8, but among them, *H. salminicola* and *H. vulgaris* have too few gene pairs and cannot be used to do 4DTv analyses. For *K. iwatai*, the genome is very unusual and cannot be used to calculate 4DTv. Because there are a lot of nearly identical gene pairs in *K. iwatai*, which might be due to redundancy instead of WGDs, so we only included 4 species in 4DTv analyses.

## Supplementary Information

**Additional file 1: Table S1.** Statistics of the Pacbio sequencing and assembly of the *Myxobolus honghuensis* genome. **Table S2.** Length distribution of the Pacbio subreads. **Table S3.** Estimated genome characteristics. **Table S4.** Clean reads mapping result. **Table S5.** Details of the gene prediction results. **Table S6.** Integration annotation file of *Myxobolus honghuensis* genes. **Table S7.** Composition of repetitive sequences in the *Myxobolus honghuensis* genome. **Table S8.** Syntenic analysis of *Myxobolus honghuensis* and 6 other cnidarian genomes. **Table S9.** Presence and absence of nuclear-encoded proteins that are targeted to mitochondria in the *Myxobolus honghuensis* proteome. **Table S10.** Summary of orthologous and paralogous gene families in *Myxobolus honghuensis* and 15 other sequenced cnidarian genomes. **Table S11.** Annotation of the unique genes in *Myxobolus honghuensis* genome. **Table S12.** Distribution of expanded and contracted gene families among the 16 cnidarian genomes. **Table S13.** Annotation of the expanded gene families in *Myxobolus honghuensis* genome. **Table S14.** Annotation of the contracted gene families in *Myxobolus honghuensis* genome. **Table S15.** Positively selected genes identified in *Myxobolus honghuensis* genome. **Table S16.** Presence and absence of 20 meiosis-related genes in the genome and transcriptome of *Myxobolus honghuensis*. **Table S17.** KEGG pathway enrichment analysis of unique genes. **Table S18.** KEGG pathway enrichment analysis of expanded and contracted genes. **Table S19.** Presence and absence of ANTP class Homeobox genes in *M. honghuensis* and other free-living cnidarians. **Table S20.** Details of sampling and sequencing. **Table S21.** Results for mapping Illumina genome sequences to the common carp genome (NCBI: GCF_000951615.1) using SOAP. **Table S22.** Statistics of the bacterial genome assembly. **Table S23.** Information of the species used in comparative genomics.

**Additional file 2: Figure S1.** K-mer distribution of survey genome sequencing reads of *Myxobolus honghuensis*. (A) 17-mer frequency percentage distribution curve of sequencing reads. (B) The product of 17-mer frequency and corresponding depth percentage distribution curve of sequencing reads. K-mers (K=17) were extracted from the paired-end library with an insert size of 500 bp. The total 17-mer count is 17,472,358,830. The peak 17-mer depth was 85, and the genome size was calculated as is 17,472,358,830/85 = 205.6 Mb. **Figure S2.** GO classification of unique genes in *Myxobolus honghuensis*. **Figure S3.** KEGG pathway analysis of unique genes in *Myxobolus honghuensis*. **Figure S4.** The biological process GO enrichment graph of unique genes in *Myxobolus honghuensis*. The redder the rectangle, the higher the degree of enrichment. **Figure S5.** The cellular component GO enrichment graph of unique genes in *Myxobolus honghuensis*. The redder the rectangle, the higher the degree of enrichment. **Figure S6.** The molecular function GO enrichment graph of unique genes in *Myxobolus honghuensis*. The redder the rectangle, the higher the degree of enrichment. **Figure S7.** GO classification of expanded genes in *Myxobolus honghuensis*. **Figure S8.** KEGG pathway analysis of expanded genes in *Myxobolus honghuensis*. **Figure S9.** The biological process GO enrichment graph of expanded genes in *Myxobolus honghuensis*. The redder the rectangle, the higher the degree of enrichment. **Figure S10.** The cellular component GO enrichment graph of expanded genes in *Myxobolus honghuensis*. The redder the rectangle, the higher the degree of enrichment. **Figure S11.** The molecular function GO enrichment graph of expanded genes in *Myxobolus honghuensis*. The redder the rectangle, the higher the degree of enrichment. **Figure S12.** GO classification of contracted genes in *Myxobolus honghuensis*. **Figure S13.** KEGG pathway analysis of contracted genes in *Myxobolus honghuensis*. **Figure S14.** The biological process GO enrichment graph of contracted genes in *Myxobolus honghuensis*. The redder the rectangle, the higher the degree of enrichment. **Figure S15.** The cellular component GO enrichment graph of contracted genes in *Myxobolus honghuensis*. The redder the rectangle, the higher the degree of enrichment. **Figure S16.** The molecular function GO enrichment graph of contracted genes in *Myxobolus honghuensis*. The redder the rectangle, the higher the degree of enrichment. **Figure S17.** Phylogenetic tree of *Myxobolus honghuensis* (in bold) and 15 other species based on maximum likelihood analysis of a concatenated alignment of widespread single-copy protein sequences (51 genes including 11,323 amino acids)

## Data Availability

Data generated and analyzed during this study are included in the published article, its additional files, and publicly available repositories. The raw reads of the *M. honghuensis* transcriptome sequencing, Illumina genome sequencing, and PacBio genome sequencing have been deposited at the NCBI Short Read Archive with the project accession numbers PRJNA779260 [154], PRJNA778632 [155], and PRJNA779846 [156]. The transcriptome assembly has been deposited at DDBJ/EMBL/GenBank under the accession GJPJ00000000 [157]. The Illumina genome assembly have been deposited in the Genome Warehouse (GWH) in National Genomics Data Center (NGDC) under the accession GWHBFXM00000000 [158]. The PacBio genome assembly and related annotation information have been deposited in the GWH in NGDC under the accession GWHBFXL00000000 [159] and Harvard Dataverse: 10.7910/DVN/INLEPM [160]. Alignment and maximum likelihood tree have been deposited in the TreeBASE repository: http://purl.org/phylo/treebase/phylows/study/TB2:S28997 [161].

## Abbreviations

ABC: ATP-binding cassette
ANTP: Antennapedia
CLEC: C-type lectins
ECM: Extracellular matrix
kb: kilobase
LDLRs: Low-density lipoprotein receptors
LTRs: Long terminal repeats
Mb: Megabase
MFS: Major facilitator superfamily
Mt: Mitochondrial
Mya: Million years ago
PSDs: Postsynaptic densities
rRNA: Ribosomal RNA
TE: Transposable element
TIRs: Terminal inverted repeats
WGDs: Whole-genome duplications
4DTv: Fourfold synonymous third-codon transversion
