# Supplementary material for: A myxozoan genome reveals mosaic evolution in a parasitic cnidarian
Source: BMC Biol. 2022 Feb 18;20:51. doi: 10.1186/s12915-022-01249-8 (PMC8855578; doi:10.1186/s12915-022-01249-8)
Supplement: Supplementary file 2 — Additional file 2: Figure S1. K-mer distribution of survey genome sequencing reads of Myxobolus honghuensis. (A) 17-mer frequency percentage distribution curve of sequencing reads. (B) The product of 17-mer frequency and corresponding depth percentage distribution curve of sequencing reads. K-mers (K=17) were extracted from the paired-end library with an insert size of 500 bp. The total 17-mer count is 17,472,358,830. The peak 17-mer depth was 85, and the genome size was calculated as is 17,472,358,830/85 = 205.6 Mb. Figure S2. GO classification of unique genes in Myxobolus honghuensis. Figure S3. KEGG pathway analysis of unique genes in Myxobolus honghuensis. Figure S4. The biological process GO enrichment graph of unique genes in Myxobolus honghuensis. The redder the rectangle, the higher the degree of enrichment. Figure S5. The cellular component GO enrichment graph of unique genes in Myxobolus honghuensis. The redder the rectangle, the higher the degree of enrichment. Figure S6. The molecular function GO enrichment graph of unique genes in Myxobolus honghuensis. The redder the rectangle, the higher the degree of enrichment. Figure S7. GO classification of expanded genes in Myxobolus honghuensis. Figure S8. KEGG pathway analysis of expanded genes in Myxobolus honghuensis. Figure S9. The biological process GO enrichment graph of expanded genes in Myxobolus honghuensis. The redder the rectangle, the higher the degree of enrichment. Figure S10. The cellular component GO enrichment graph of expanded genes in Myxobolus honghuensis. The redder the rectangle, the higher the degree of enrichment. Figure S11. The molecular function GO enrichment graph of expanded genes in Myxobolus honghuensis. The redder the rectangle, the higher the degree of enrichment. Figure S12. GO classification of contracted genes in Myxobolus honghuensis. Figure S13. KEGG pathway analysis of contracted genes in Myxobolus honghuensis. Figure S14. The biological process GO enrichment graph of contr [file 12915_2022_1249_MOESM2_ESM.docx]

**Supplementary Information for**

**A myxozoan genome reveals mosaic evolution in a parasitic cnidarian**

Qingxiang Guo^1,2†^, Stephen D. Atkinson^3†^, Bin Xiao^1,2^, Yanhua Zhai^1,2^, Jerri L. Bartholomew^3^, Zemao Gu (顾泽茂)^1,2*^

^*^Correspondence: [guzemao@mail.hzau.edu.cn](mailto:guzemao@mail.hzau.edu.cn)

^†^Qingxiang Guo and Stephen D. Atkinson contributed equally to this work.

^1^Department of Aquatic Animal Medicine, College of Fisheries, Huazhong Agricultural University, Wuhan 430070, PR China

^2^Hubei Engineering Technology Research Center for Aquatic Animal Diseases Control and Prevention, Wuhan 430070, PR China

^3^Department of Microbiology, Oregon State University, Corvallis, OR 97331


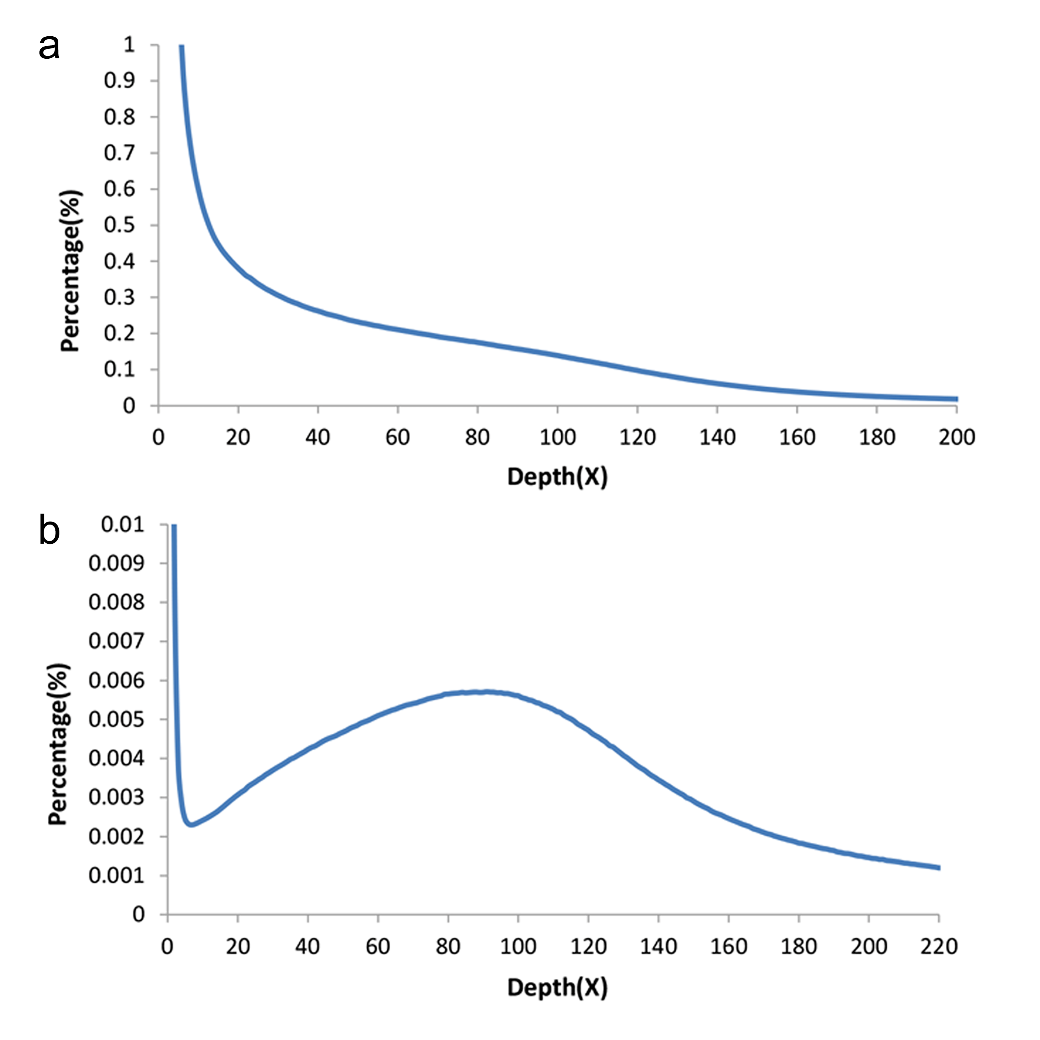


**Fig. S1**. K-mer distribution of survey genome sequencing reads of *Myxobolus honghuensis*. (A) 17-mer frequency percentage distribution curve of sequencing reads. (B) The product of 17-mer frequency and corresponding depth percentage distribution curve of sequencing reads. K-mers (K=17) were extracted from the paired-end library with an insert size of 500 bp. The total 17-mer count is 17,472,358,830. The peak 17-mer depth was 85, and the genome size was calculated as is 17,472,358,830/85 = 205.6 Mb.


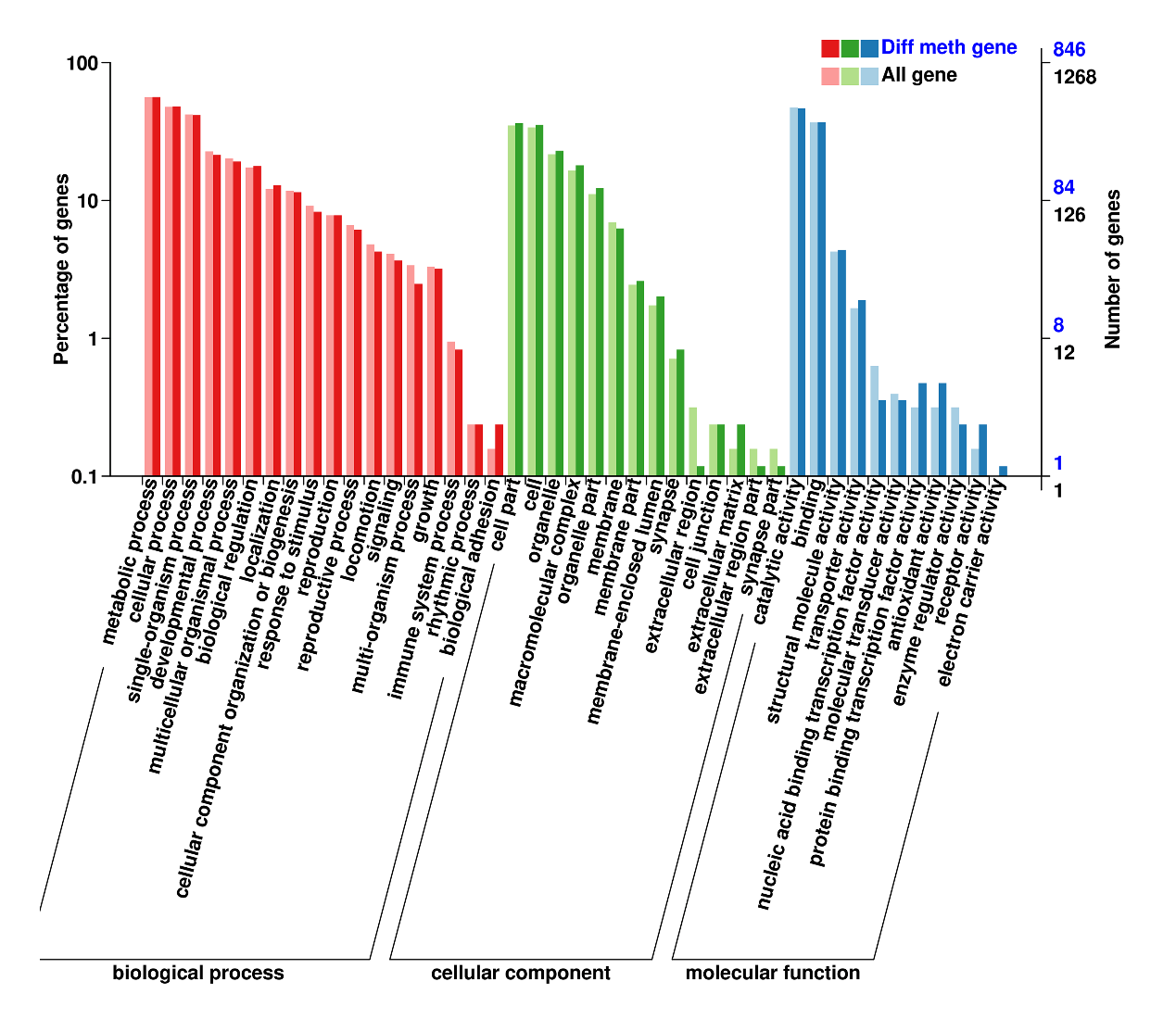


**Fig. S2**. GO classification of unique genes in *Myxobolus honghuensis*.


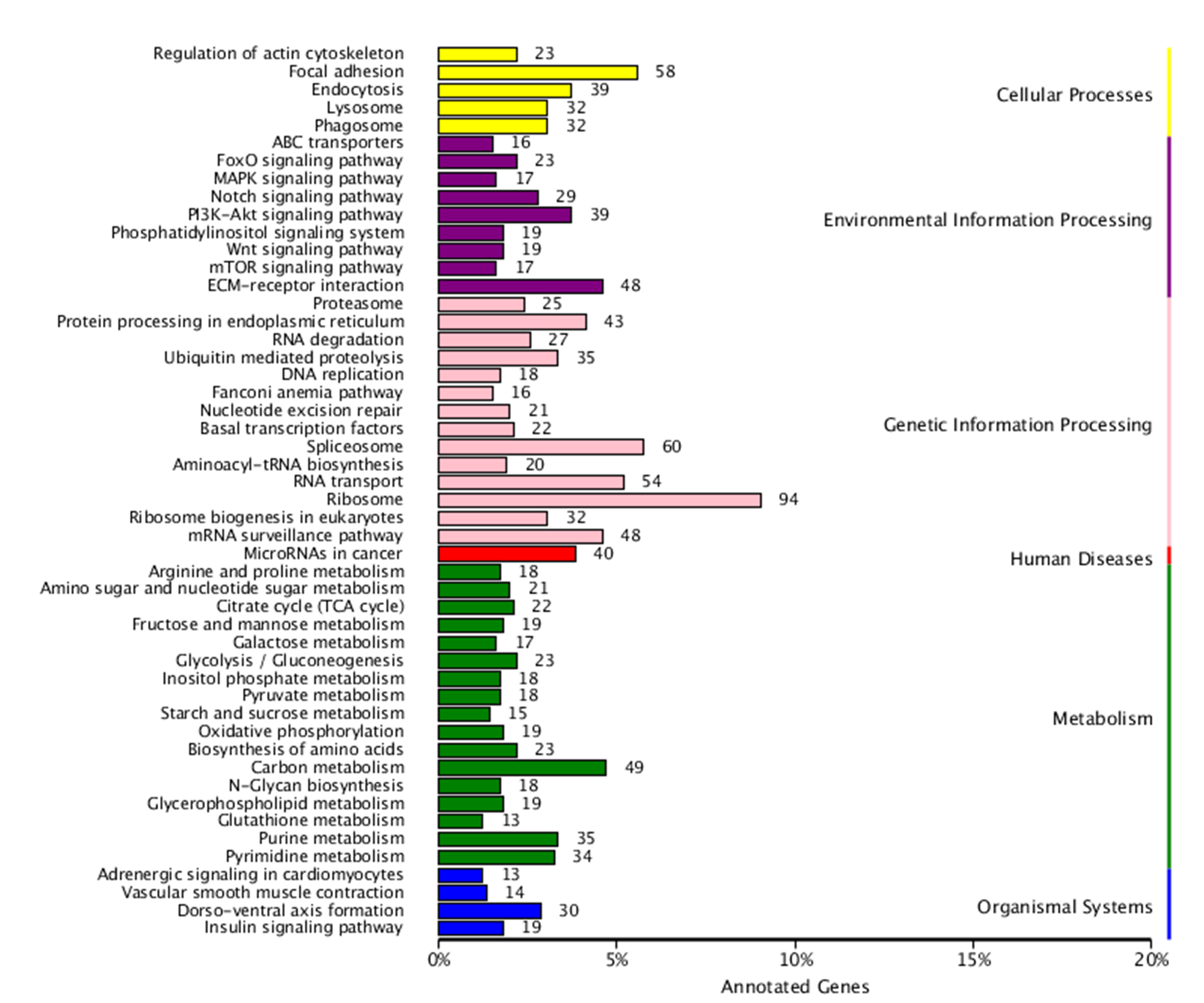


**Fig. S3**. KEGG pathway analysis of unique genes in *Myxobolus honghuensis*.


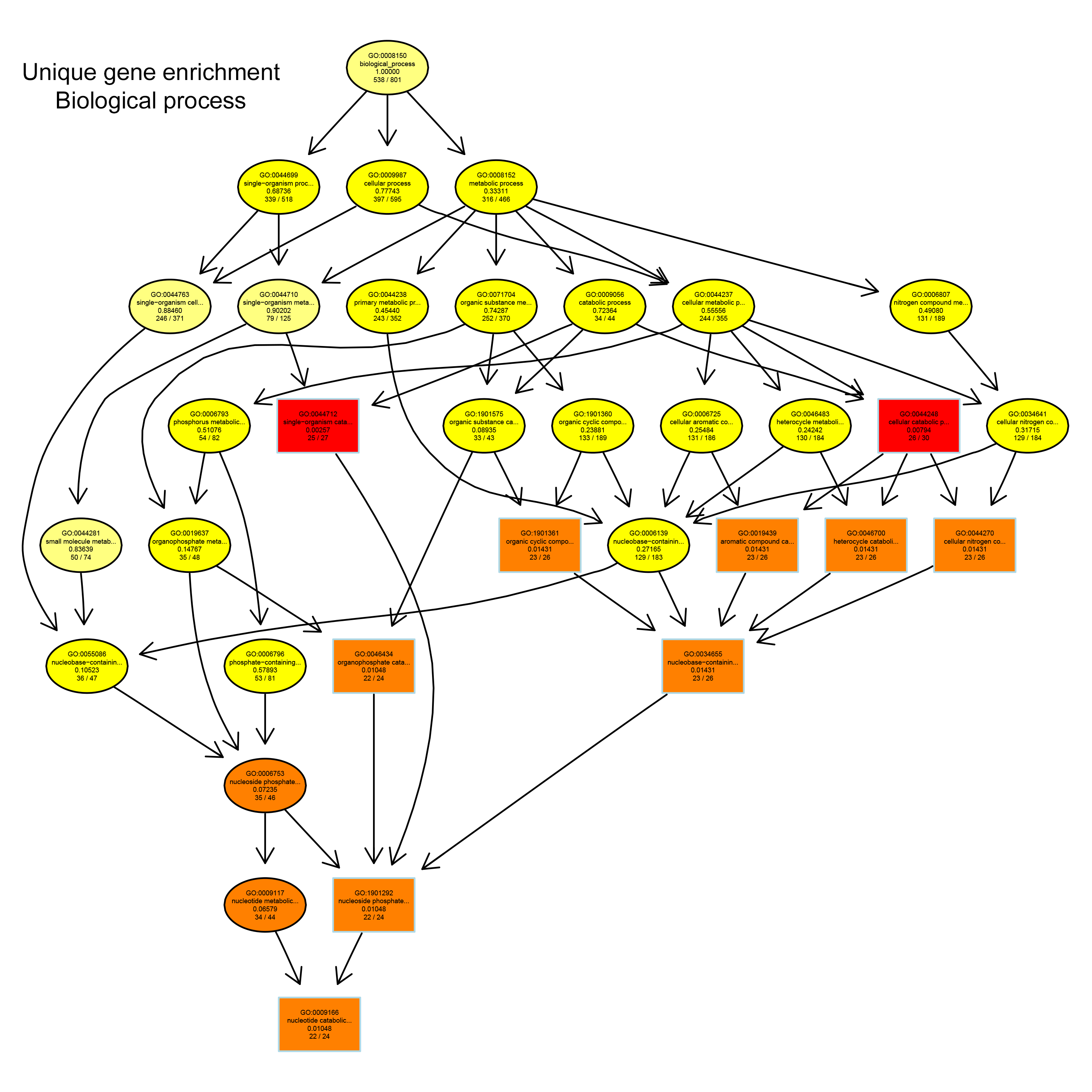


**Fig. S4**. The biological process GO enrichment graph of unique genes in *Myxobolus honghuensis*. The redder the rectangle, the higher the degree of enrichment.


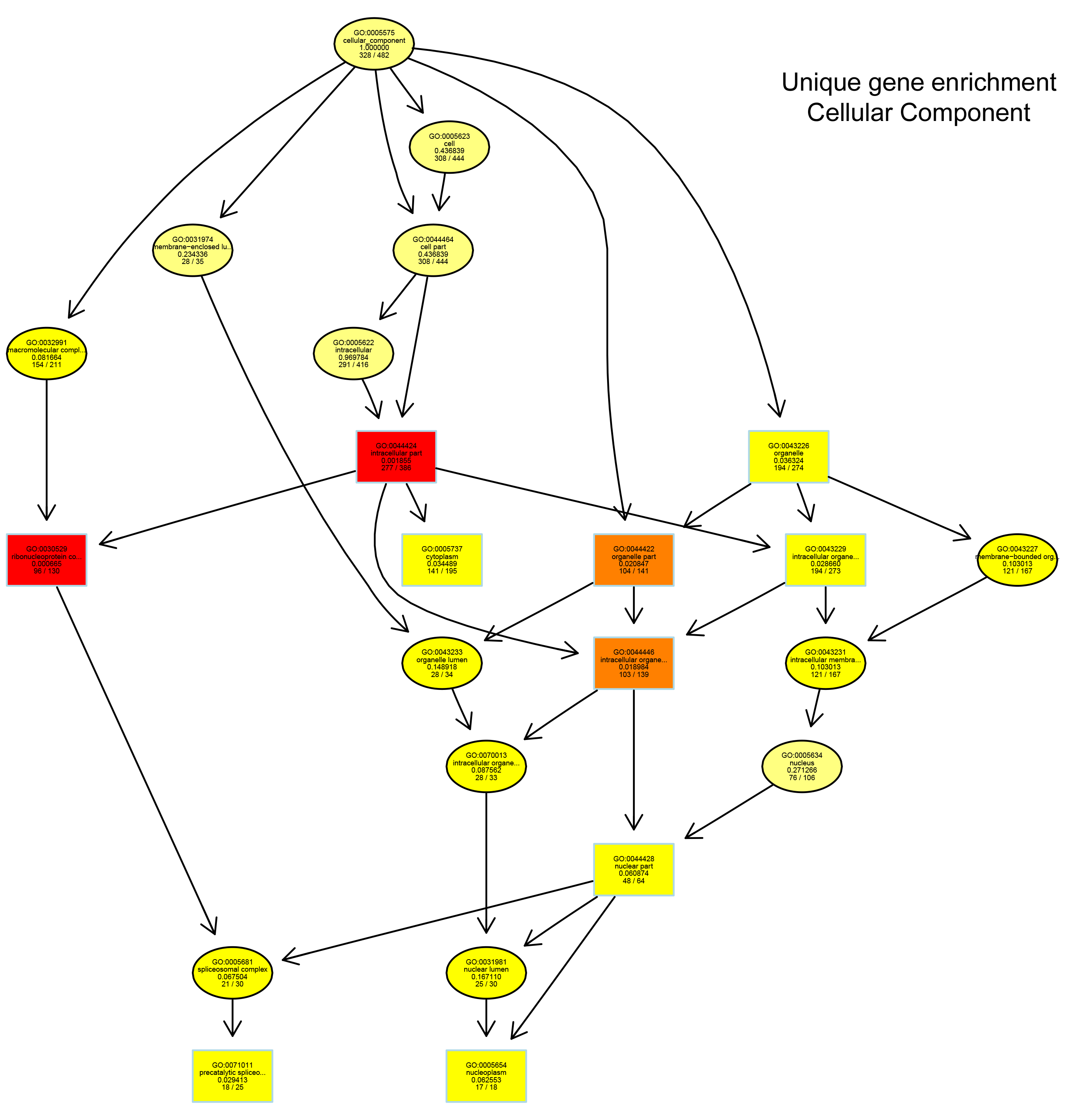


**Fig. S5**. The cellular component GO enrichment graph of unique genes in *Myxobolus honghuensis*. The redder the rectangle, the higher the degree of enrichment.


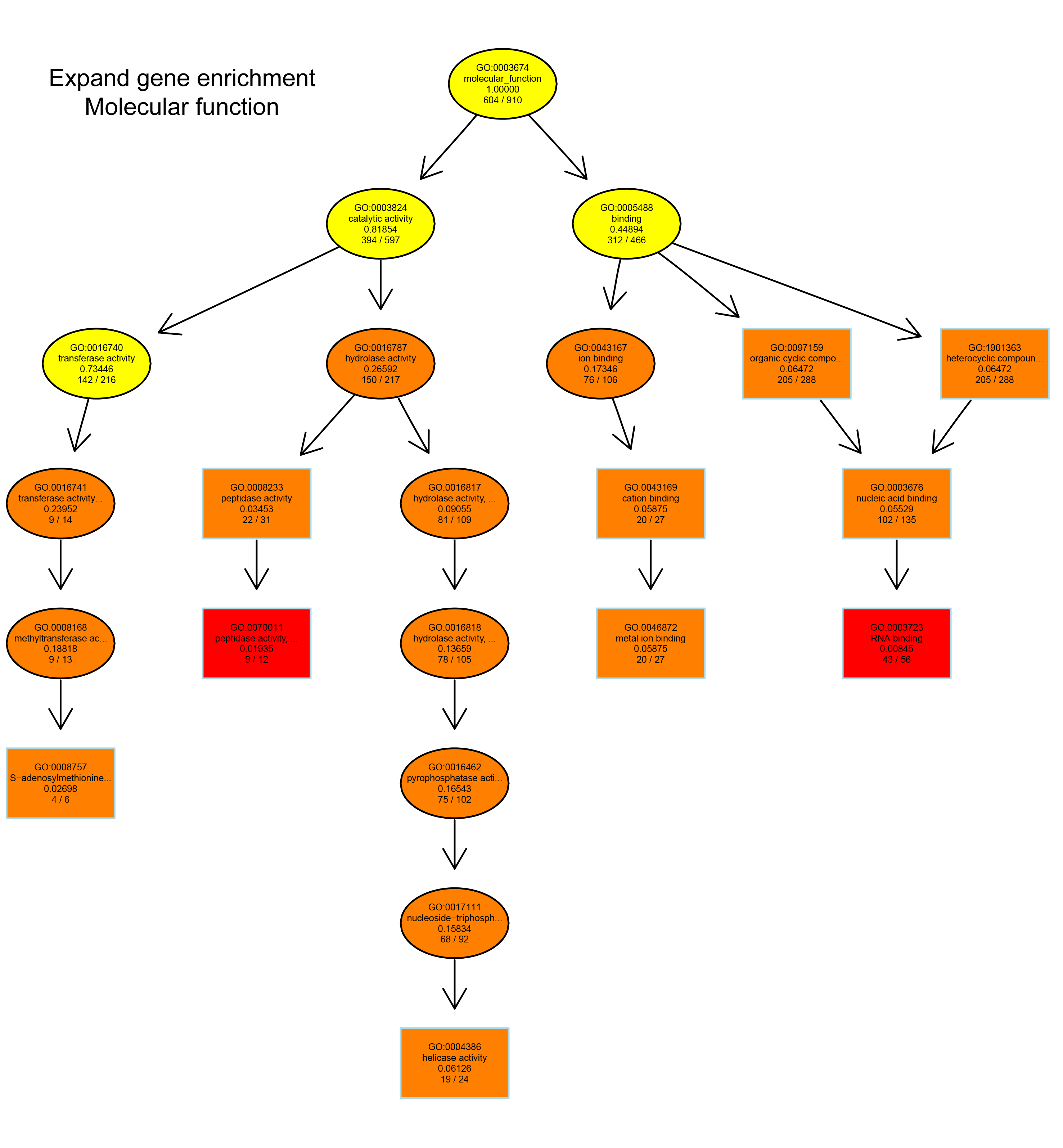


**Fig. S6**. The molecular function GO enrichment graph of unique genes in *Myxobolus honghuensis*. The redder the rectangle, the higher the degree of enrichment.


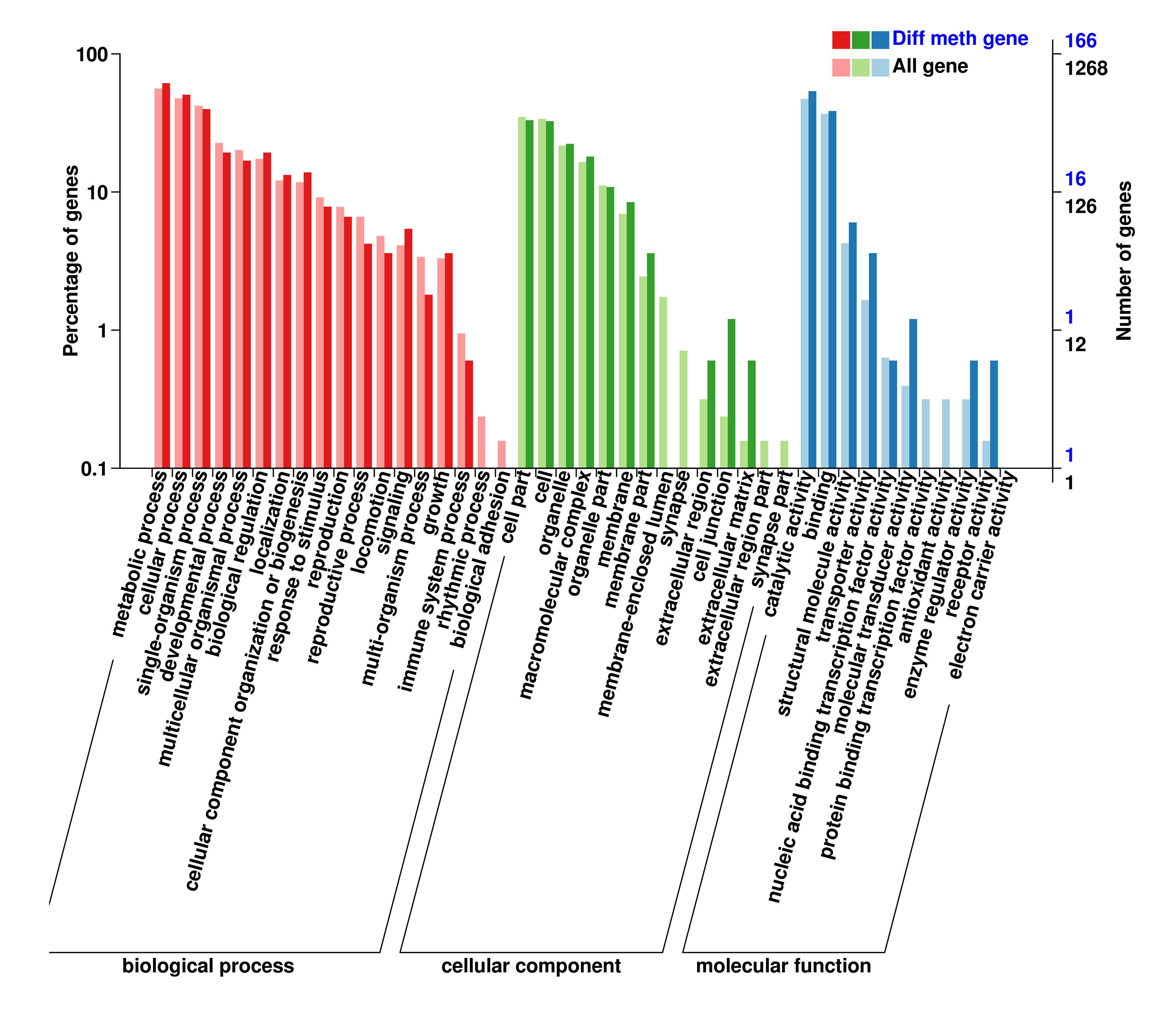


**Fig. S7**. GO classification of expanded genes in *Myxobolus honghuensis*.


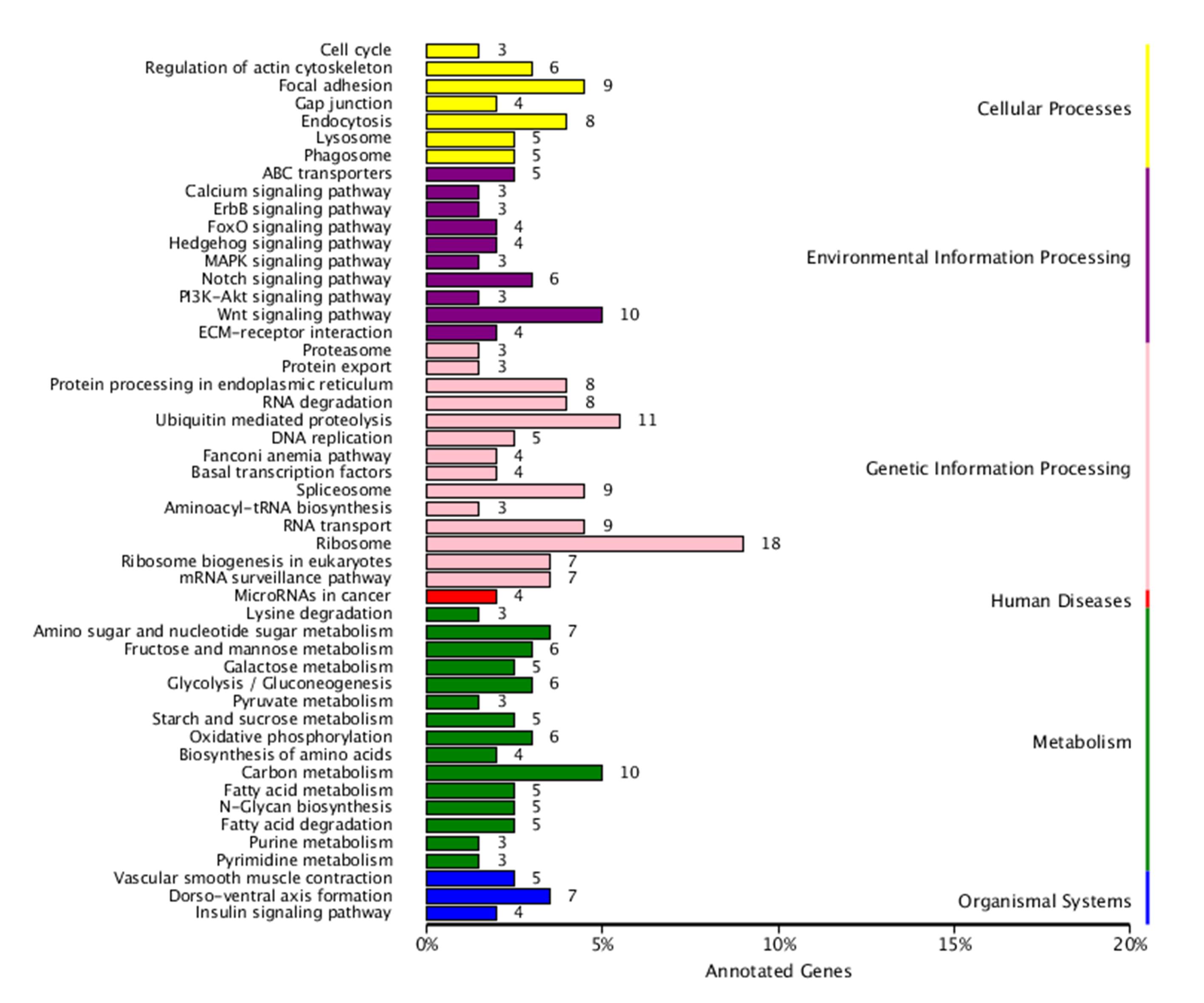


**Fig. S8**. KEGG pathway analysis of expanded genes in *Myxobolus honghuensis*.


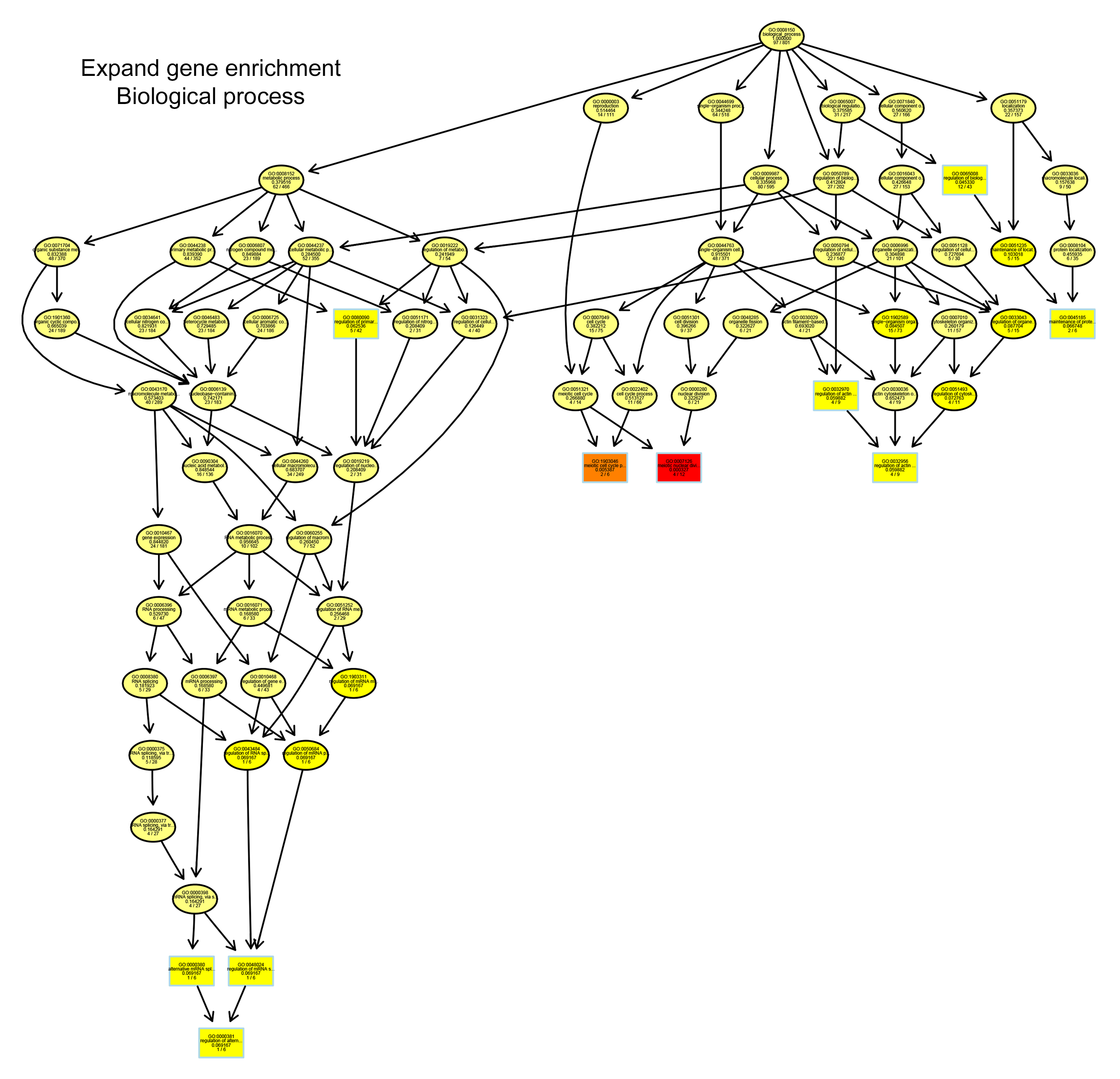


**Fig. S9**. The biological process GO enrichment graph of expanded genes in *Myxobolus honghuensis*. The redder the rectangle, the higher the degree of enrichment.


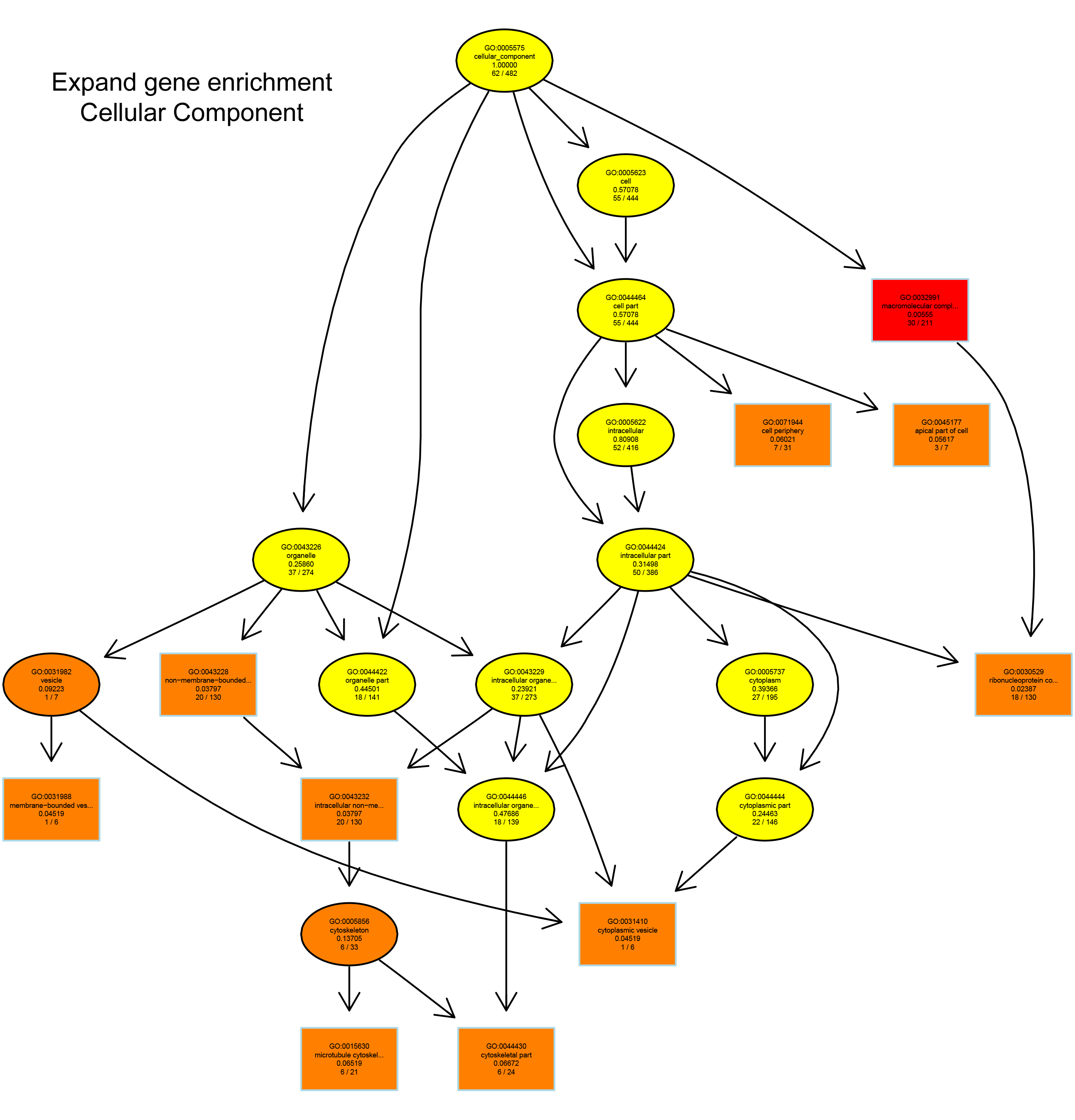


**Fig. S10**. The cellular component GO enrichment graph of expanded genes in *Myxobolus honghuensis*. The redder the rectangle, the higher the degree of enrichment.


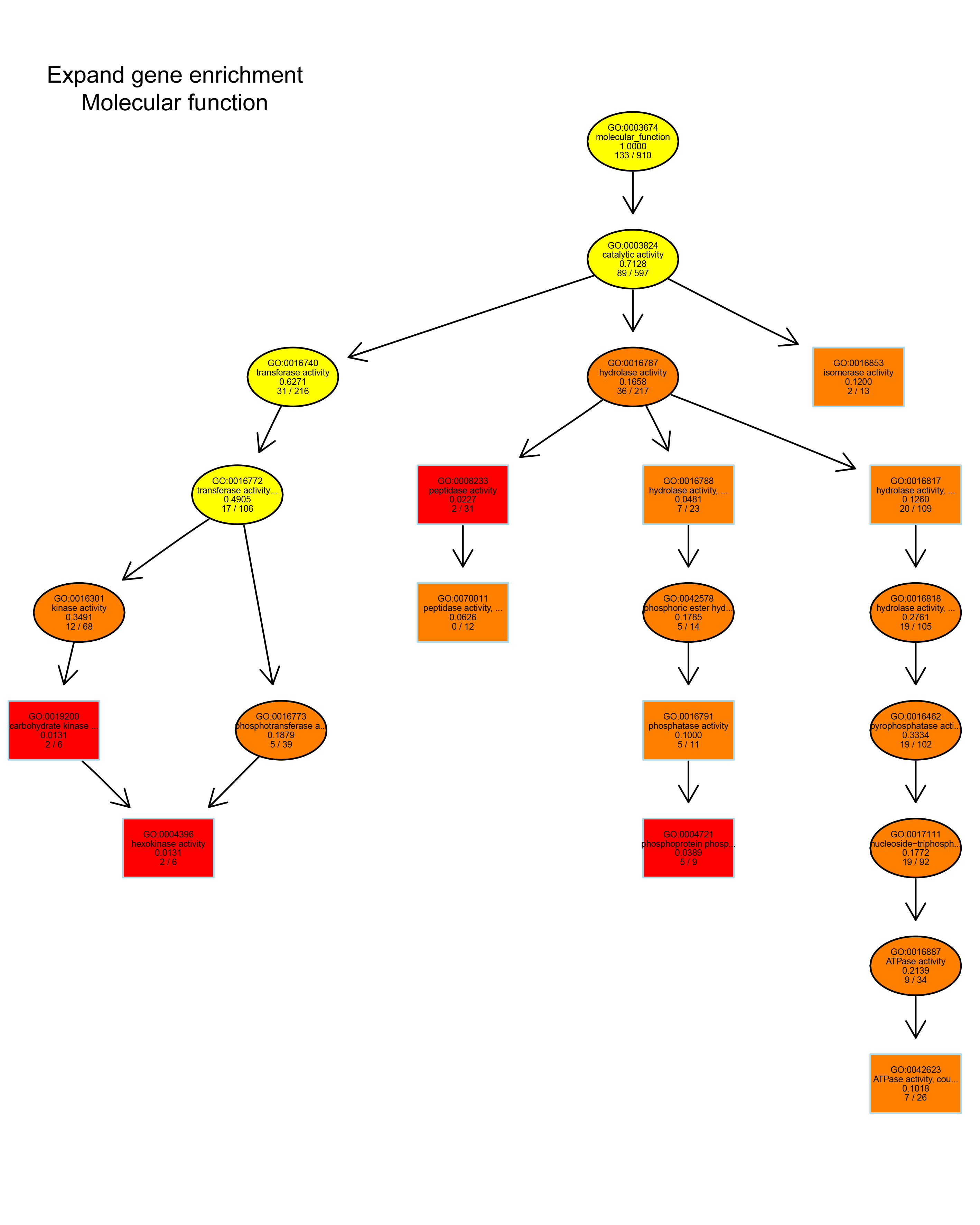


**Fig. S11**. The molecular function GO enrichment graph of expanded genes in *Myxobolus honghuensis*. The redder the rectangle, the higher the degree of enrichment.


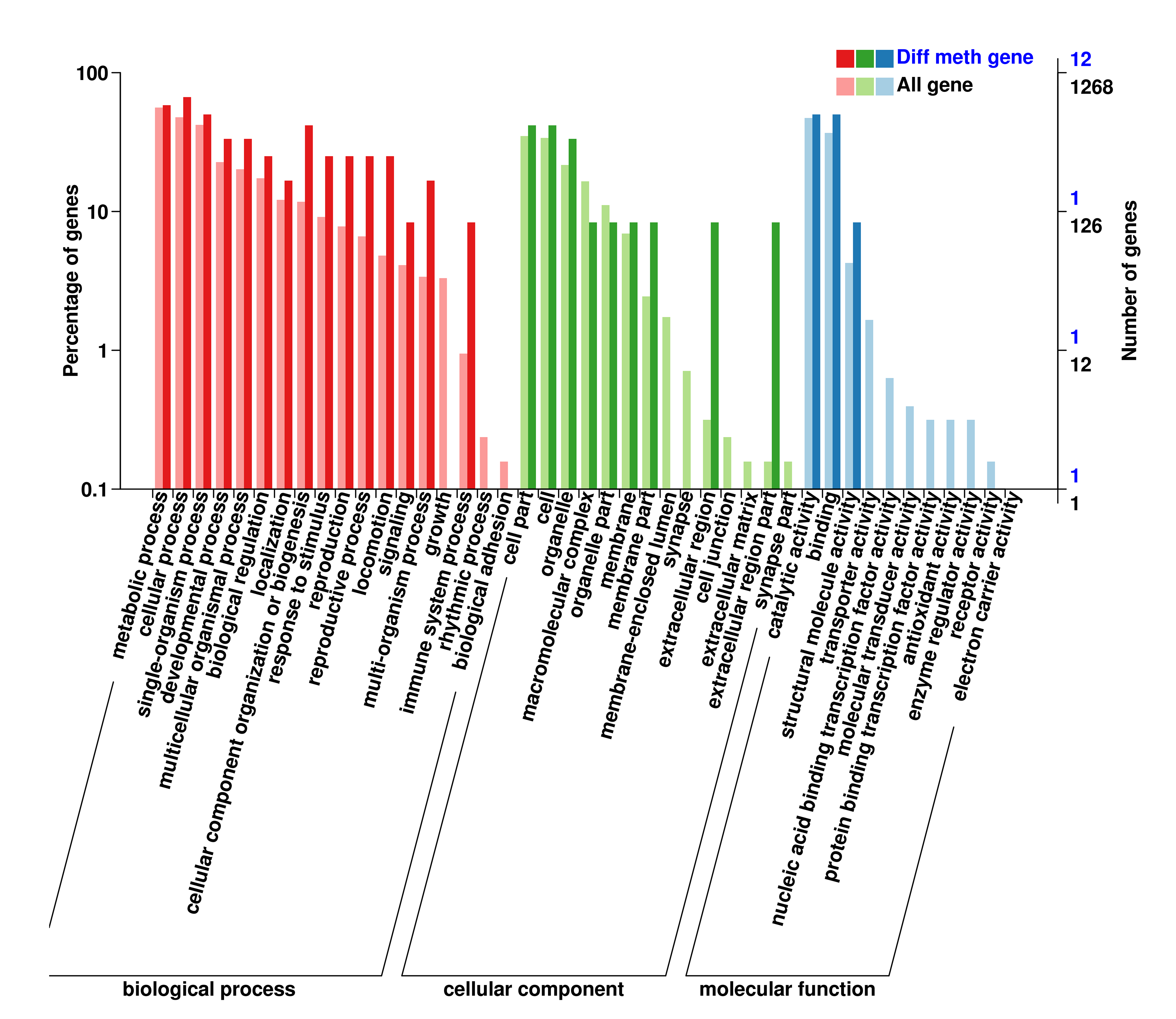


**Fig. S12**. GO classification of contracted genes in *Myxobolus honghuensis*.


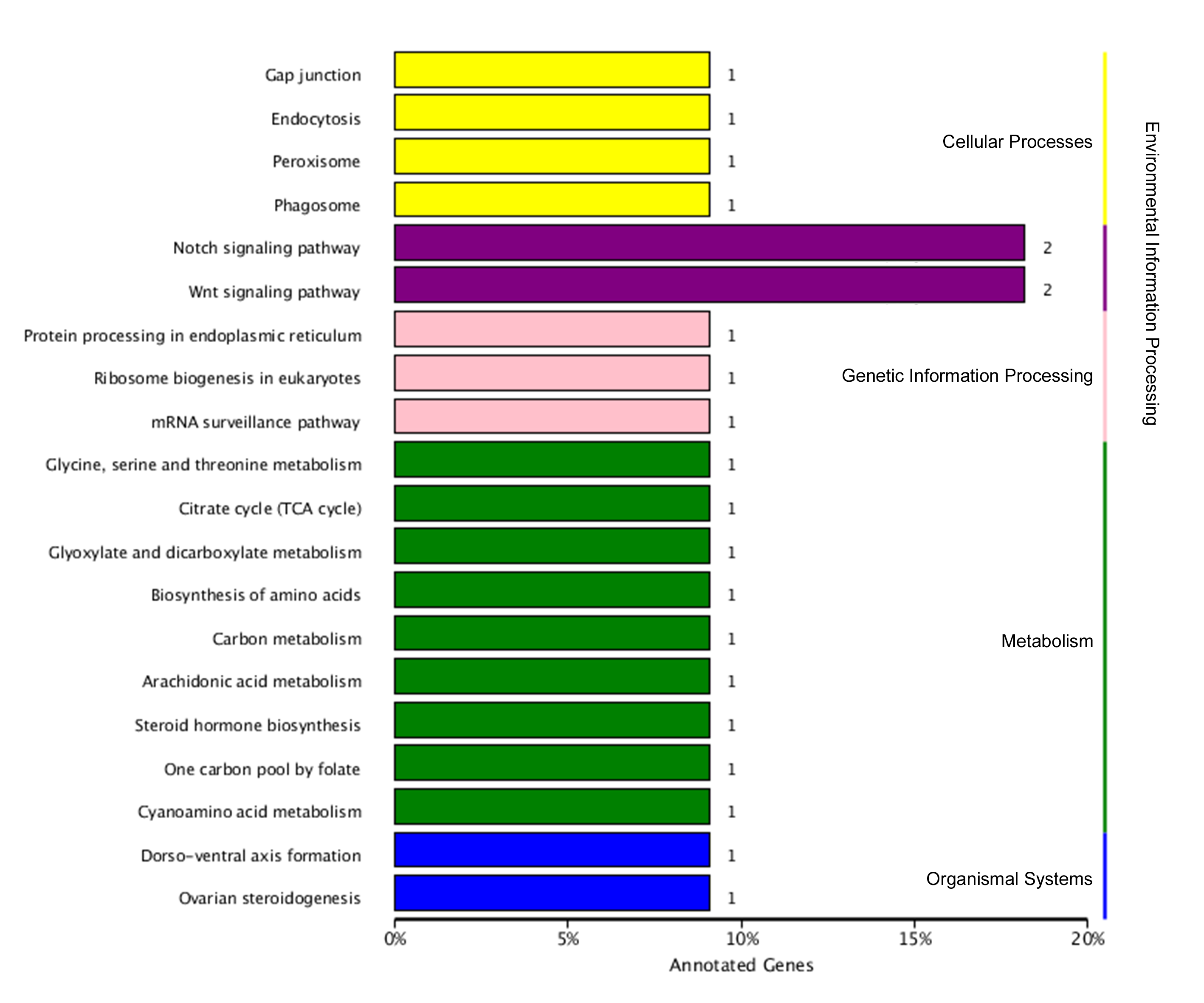


**Fig. S13**. KEGG pathway analysis of contracted genes in *Myxobolus honghuensis*.


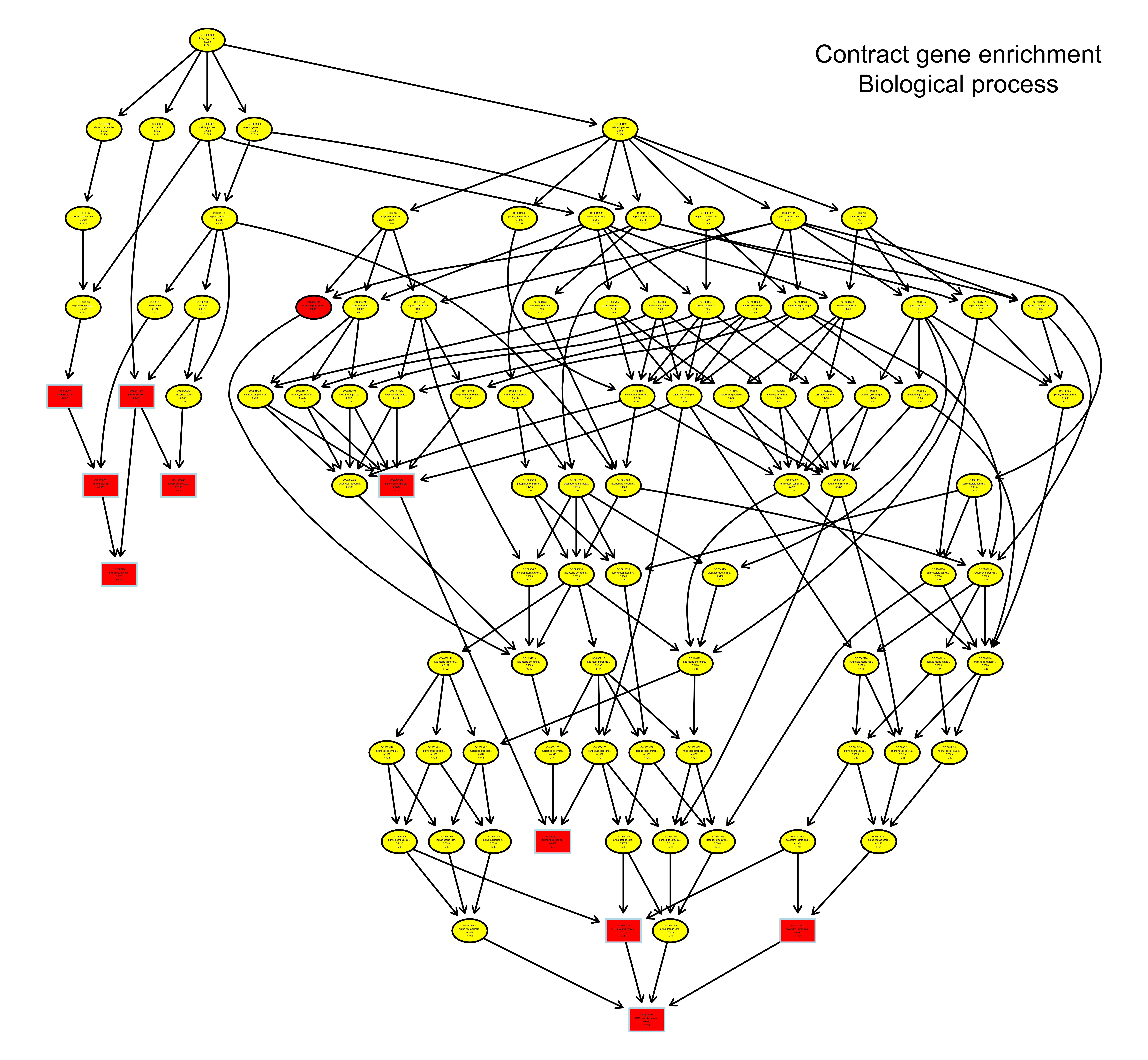


**Fig. S14**. The biological process GO enrichment graph of contracted genes in *Myxobolus honghuensis*. The redder the rectangle, the higher the degree of enrichment.


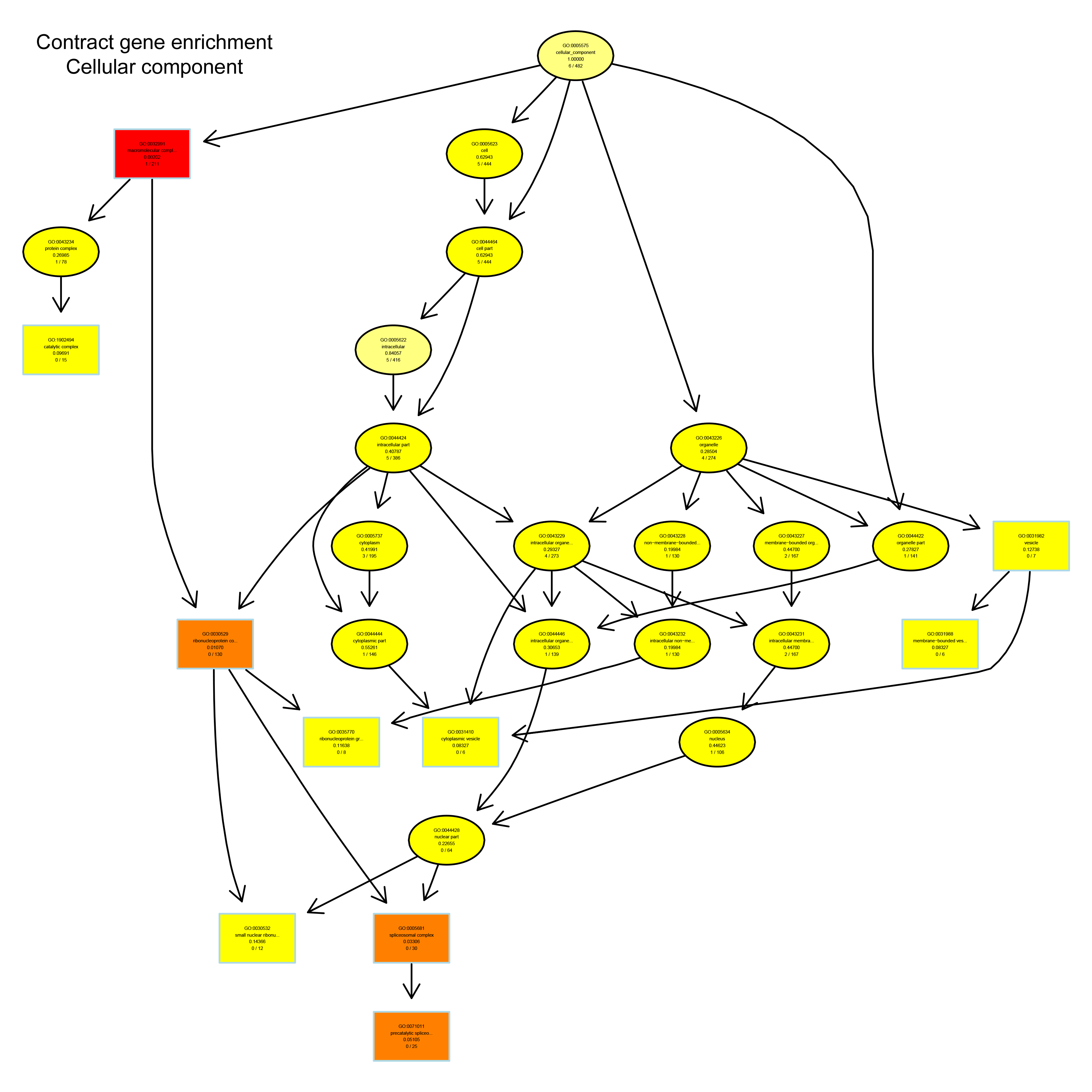


**Fig. S15**. The cellular component GO enrichment graph of contracted genes in *Myxobolus honghuensis*. The redder the rectangle, the higher the degree of enrichment.


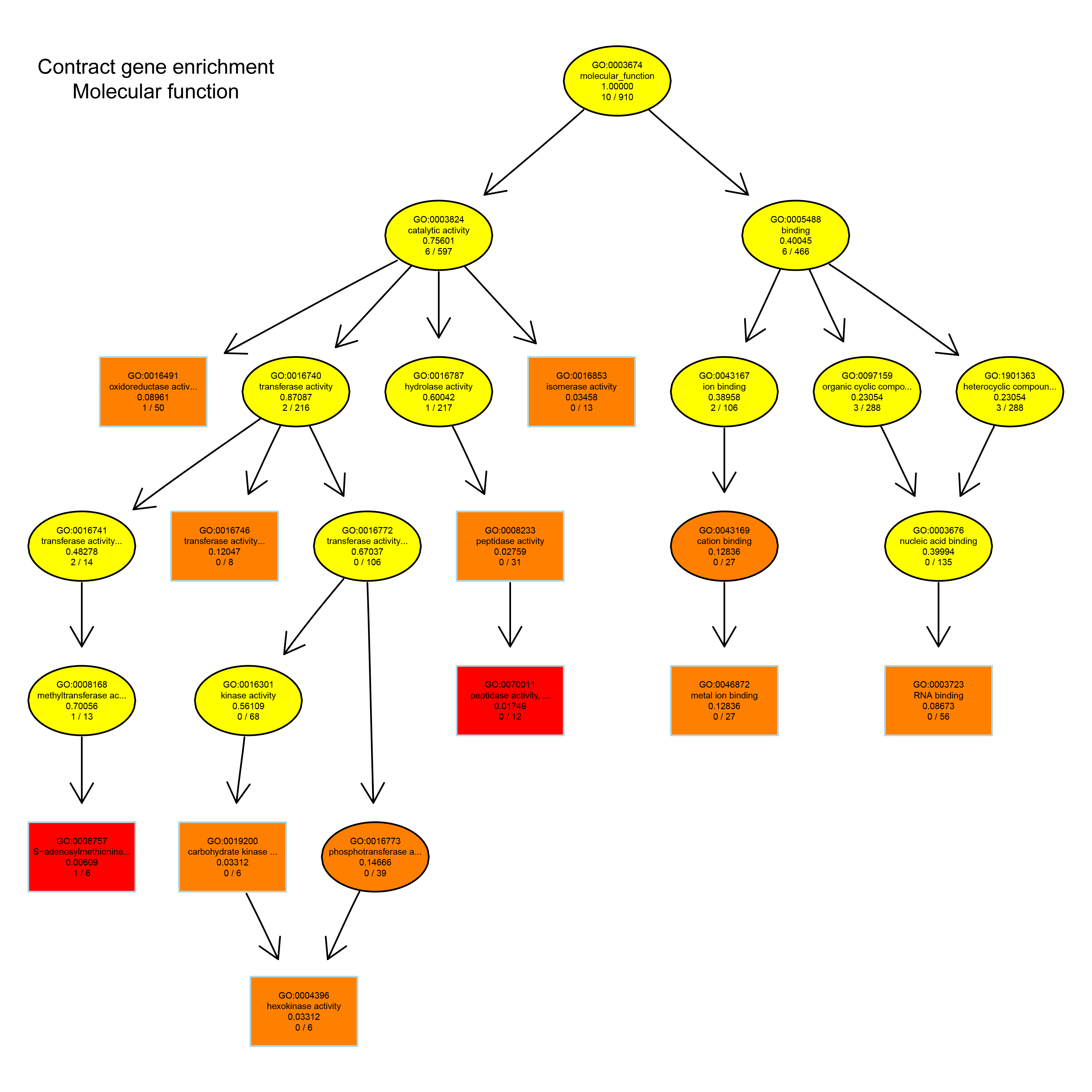


**Fig. S16**. The molecular function GO enrichment graph of contracted genes in *Myxobolus honghuensis*. The redder the rectangle, the higher the degree of enrichment.


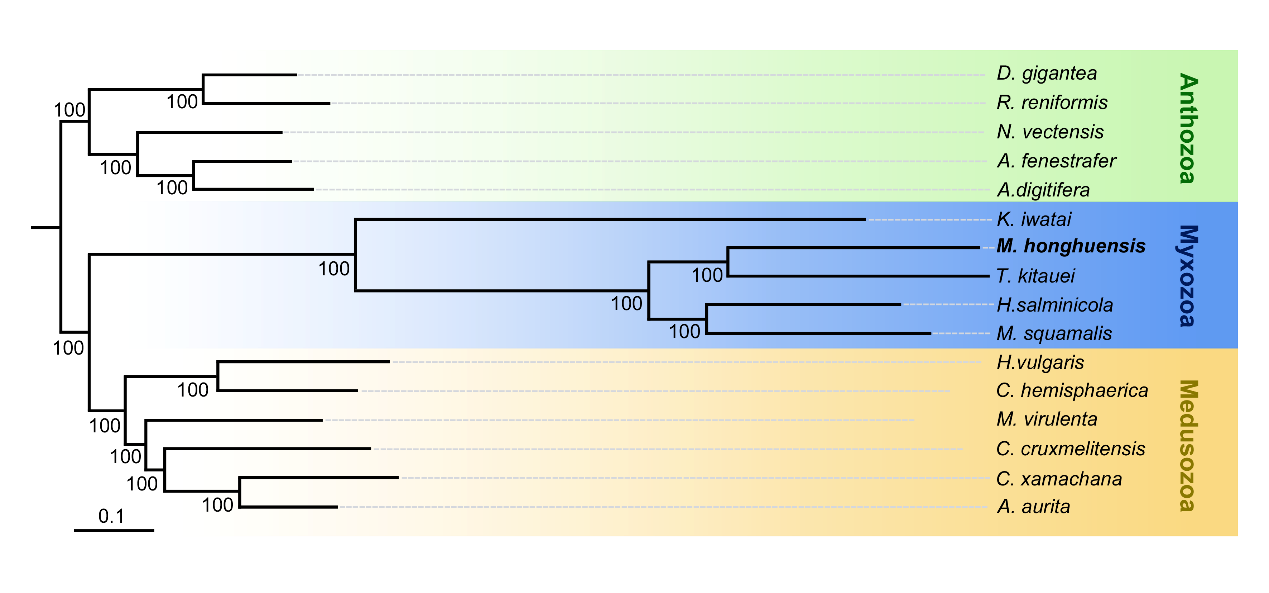


**Fig. S17.** Phylogenetic tree of *Myxobolus honghuensis* (in bold) and 15 other species based on maximum likelihood analysis of a concatenated alignment of widespread single-copy protein sequences (51 genes including 11,323 amino acids).
